# cellHarmony: cell-level matching and holistic comparison of single-cell transcriptomes

**DOI:** 10.1093/nar/gkz789

**Published:** 2019-09-16

**Authors:** Erica A K DePasquale, Daniel Schnell, Phillip Dexheimer, Kyle Ferchen, Stuart Hay, Kashish Chetal, Íñigo Valiente-Alandí, Burns C Blaxall, H Leighton Grimes, Nathan Salomonis

**Affiliations:** 1 Department of Biomedical Informatics, University of Cincinnati, Cincinnati, OH, USA; 2 Division of Biomedical Informatics, Cincinnati Children's Hospital Medical Center, Cincinnati, OH, USA; 3 Heart Institute and Center for Translational Fibrosis Research, Cincinnati Children's Hospital Medical Center, Cincinnati, OH, USA; 4 Department of Cancer Biology, University of Cincinnati, Cincinnati, OH, USA; 5 Division of Immunobiology and Center for Systems Immunology, Cincinnati Children's Hospital Medical Center, Cincinnati, OH, USA; 6 Department of Pediatrics, University of Cincinnati School of Medicine, Cincinnati, OH, USA; 7 Division of Experimental Hematology and Cancer Biology, Cincinnati Children's Hospital Medical Center, Cincinnati, OH, USA

## Abstract

To understand the molecular pathogenesis of human disease, precision analyses to define alterations within and between disease-associated cell populations are desperately needed. Single-cell genomics represents an ideal platform to enable the identification and comparison of normal and diseased transcriptional cell populations. We created cellHarmony, an integrated solution for the unsupervised analysis, classification, and comparison of cell types from diverse single-cell RNA-Seq datasets. cellHarmony efficiently and accurately matches single-cell transcriptomes using a community-clustering and alignment strategy to compute differences in cell-type specific gene expression over potentially dozens of cell populations. Such transcriptional differences are used to automatically identify distinct and shared gene programs among cell-types and identify impacted pathways and transcriptional regulatory networks to understand the impact of perturbations at a systems level. cellHarmony is implemented as a python package and as an integrated workflow within the software AltAnalyze. We demonstrate that cellHarmony has improved or equivalent performance to alternative label projection methods, is able to identify the likely cellular origins of malignant states, stratify patients into clinical disease subtypes from identified gene programs, resolve discrete disease networks impacting specific cell-types, and illuminate therapeutic mechanisms. Thus, this approach holds tremendous promise in revealing the molecular and cellular origins of complex disease.

## INTRODUCTION

Single-cell RNA-sequencing (scRNA-Seq) provides the unique ability to profile transcripts from diverse cell populations along a continuum of related or disparate cell types (1). In addition to defining known and novel cell populations, single-cell technologies can identify disease-related gene regulatory programs which underlie molecular and cellular dysfunction. While diverse single-cell experimental platforms exist to facilitate such analyses, there is an urgent need for integrated and easy-to-use computational approaches to identify discrete differences between comparable diseased and healthy cells. Given that most scRNA-Seq analyses will potentially identify dozens of cell populations, such an exercise becomes non-trivial, as distinct cell populations will have different transcriptional, cellular, pathway and gene regulatory network impacts. Furthermore, cellular and molecular differences can occur in either a cell type-specific manner or across a spectrum of related cell populations, requiring new holistic analysis solutions. Given the complexity of the analyses required to achieve these goals, automated solutions that can be applied by both experienced bioinformaticians and conventional laboratory biologists are ultimately required.

The development of workflows to provide disease-level insights requires reproducible mapping and comparison of single-cell transcriptomes across one or more samples. Two principal classes of algorithms are designed to align and compare scRNA-Seq datasets: (i) label projection and (ii) joint alignment. Label projection methods consider a reference scRNA-Seq dataset with already defined clusters as the basis for assigning those cell type annotations to new datasets. In the case of disease, the objective of such algorithms is to annotate perturbed cell states according to their most closely related normal equivalents, without considering novel cell populations observed uniquely in disease. A number of algorithms have been recently developed to perform this objective including scmap, Seurat3, conos, Garnett, CHETAH and SingleCellNet (see Table 1 for a comparison of features and methods) (2–6). Notable among these tools are conos and Seurat, which enable the downstream comparison of cell-populations using differential expression analyses. A potential limitation of this analysis for conos is that two individual datasets cannot be compared by this method, as it requires biological replicate scRNA-Seq experiments for analysis with DESeq2. While Seurat enables the direct comparison of cells within the same population across conditions (differential expression analysis), it currently provides no means to integrate these results over potentially dozens of cell populations or prioritize impacts within specific cell types to obtain systems-level insights.

**Table 1.** Comparison of features present in label projection and joint-alignment programs

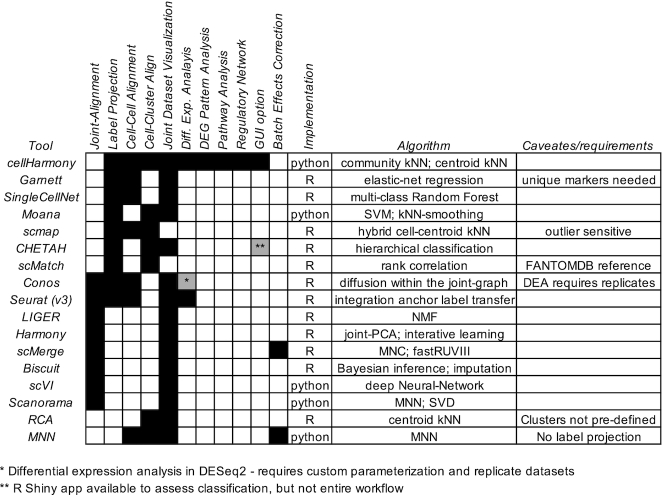

In contrast to label projection, joint-alignment methods simultaneously align similar cells into to common or distinct clusters independent of batch, donor, or other technical effects. Such tools include conos and Seurat 3 (which perform both label projection and joint-alignment), Biscuit, LIGER, Scanorama, scMerge, scVI and Harmony (Table 1) (7–12). As such, these tools can identify similar cell populations that occur in independent datasets, highly distinct cell populations that are unique to one dataset or cell type which potentially represent divergent lineage programs. While in principle such methods would appear to be a more appropriate for comparing healthy and disease states, there are a number of important caveats that can potentially limit the use of joint-alignment methods over those for label projection. Firstly, label projection methods are designed to explicitly align disease cell types to appropriately similar healthy states, whereas joint-clustering methods will often produce overly specialized clusters in which separate disease and healthy clusters are obtained for the same cell type (13–15). In such cases, no direct correspondence to diseased cell states and normal will be provided, hindering their direct comparison. Second, healthy references can include curated cell states or clusters, in which cell types have been initially defined, rather than redefining such cell populations through joint clustering. Indeed, it is most appropriate to annotate cell populations in healthy rather than disease, in which known cell type gene expression programs are have already been defined. Thirdly, for studies in which new samples such as diseased tissues are accrued over time, it is challenging to continuously re-annotate datasets with the addition of new samples using joint-alignment, which inherently rederives clusters in each new analysis. Although label projection remains a potentially powerful approach to compare scRNA-Seq datasets using unperturbed references, several challenges remain. First, it is unclear whether disease cell states contain core cell-type identity programs to enable accurate alignment to healthy references. Once aligned, accurate means for automated gene expression comparison are needed. Finally, no existing single-cell approaches organize and order gene expression changes to highlight differences that are unique to one cell type or shared across multiple related or dissimilar cell types.

**Figure 1.**
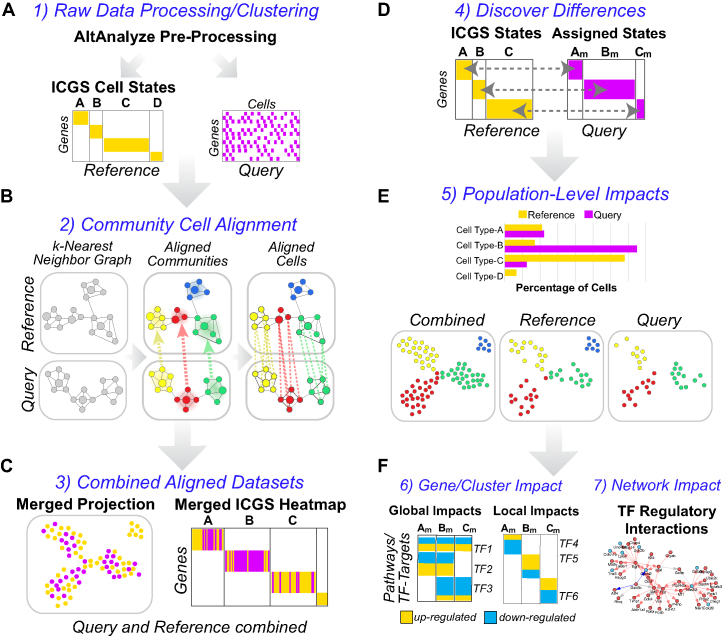
Integrated and automated holistic discovery of cell population-specific perturbations. Illustrated automated workflow of the cellHarmony analysis pipeline. (**A**) Small and large (>100k cell) scRNA-Seq datasets can be pre-processed in the parent software AltAnalyze (expression scaling, outlier exclusion) for the query and reference dataset. Raw data can be input from multiple file formats (Methods). Both query and reference can be individual samples or combined collections (joined and analyzed or merged post unsupervised analysis with the cellHarmonyMerge function). The default unsupervised clustering population prediction method in AltAnalyze is ICGS version 2.0 (ICGS-NMF output), which provides different input options (population marker genes or guide-gene correlated results). (**B**) Alignment of individual cells from the query to the reference is accomplished through community clustering of each dataset, following the creation of a k-nearest neighbor graph in each dataset. Prior to matching cells, Louvain partitions (cluster centroids) are matched between datasets to define the nearest neighborhoods (Pearson coefficient). Labels provided for the reference clusters (stem cell, macrophage…) can be provided as alternatives to cluster labels (c1,c2,c3…), based on the ICGS cluster cell-type predictions (curated by the user). (**C**) Query cells that meet a minimum correlation threshold (Pearson coefficient > 0.4, default) are placed adjacent to their aligned reference cell in the ICGS heatmap and projected into a common UMAP coordinate space to determine the mixing of cells and qualitative transcriptomic differences. (**D**) Quantitative transcriptome differences between query and reference cells are computed using a multi-step differential expression analysis between matched cell clusters and (**E**) population proportion differences reported (Fishers Exact test). (**F**) Gene-level statistical differences are used to identify the most similar impacted cell clusters (co-regulated), defined by comparing the frequency of genes similar patterns of regulation from a binarized *P*-value and fold-change matrix. Genes with expression best described by global regulation (impacted across all cell populations), co-regulated clusters (regional) or that are most restricted in their differences to specific cell clusters (local) are ordered statistically in a combined heatmap (left). Integrated with this heatmap are statistically enriched pathways and transcription-factor targets to determine the perturbation-specific impacts along a continuum of related and distinct cell populations. Putative transcriptional regulatory and curated protein-protein interaction networks are derived from each cell population or co-regulated comparison to identify likely regulators and their targets among these impacted populations (right).

Herein, we describe a new approach called cellHarmony, which provides the unique ability to obtain a unified systems-level view of molecular, cellular, pathway, and network-level differences between all aligned query and reference cell populations in an automated manner. This workflow performs three essential steps: (i) label projection of one scRNA-Seq dataset onto another, (ii) pairwise molecular and cellular comparisons of all aligned cell types and (iii) systems-level functional and regulatory analyses (Figure 1). This program is implemented as stand-alone alignment python package and is integrated into the software AltAnalyze for more streamlined scRNA-Seq analysis (1,16). We observe that in comparable disease and normal datasets, the cellular-identity programs (core genes) are retained, providing an appropriate reference for secondary comparison analyses. For label projection, cellHarmony creates a k-nearest neighbor graph of each sample to match similar sample communities (Louvain clustering) and then directly matches the cells within those communities. This strategy enables the fast alignment of similar small communities without biasing the alignment to outliers. When compared to other label projection approaches, cellHarmony results in improved alignment accuracy. Once aligned, clusters or cell-type names from one dataset can be mapped onto another and placed within a continuum of differentiation. These data are integrated and jointly visualized in multiple formats. To identify the precise impact of genetic, chemical, disease, or other perturbation, cellHarmony extracts cell type-specific differences (cell frequency and gene expression), finds, organizes, and visualizes co-regulated cell populations, examines the pathway-level impact on distinct gene modules, and produces putative gene-regulatory networks based on prior knowledge. Through seamless integration within AltAnalyze, users can jointly perform the unsupervised analysis of extremely large scRNA-Seq datasets using the algorithm Iterative Clustering and Guide-gene Selection (ICGS, version 2) prior to alignment, with little to no required bioinformatics expertise (17). Automation becomes a necessary step in these analyses, where dozens of cell populations are likely present in large single-cell RNA-seq datasets. Using this approach, we are able to effectively recapitulate previously observed disease genetic and cellular observations from models of cardiac ischemia and Acute Myeloid Leukemia (AML), determine the developmental origins of malignancy, identify novel prognostic disease biomarkers in AML, determine the cellular specificity of previously described drug targets and propose novel gene regulatory networks in these diseases.

## MATERIALS AND METHODS

### Algorithm description

#### Implementation and requirements

cellHarmony is compatible with Python 2.7 and is distributed as dedicated python source code for the community clustering alignment algorithm (https://github.com/AltAnalyze/cellHarmony-Align) and as a component of the software AltAnalyze (https://github.com/nsalomonis/altanalyze). AltAnalyze is an easy-to-use data analysis toolkit that provides both automated workflows (e.g. RNA-Seq raw data processing, alternative splicing, scRNA-Seq unsupervised analysis, microarray analysis) and separate à la cart analyses through the command-line and a dedicated graphical-user-interface. This code is supported for Linux, Mac, and Windows, using computers with a minimum of 8GB of RAM (16GB+ recommended). Additional documentation, optional R pre-processing scripts (for Seurat input references), and tutorial videos are available from: http://www.altanalyze.org/cellHarmony. When run through AltAnalyze, cellHarmony works seamlessly with the embedded unsupervised analysis software, Iterative Clustering and Guide-gene Selection (ICGS) version 2 as input (1,17). To run cellHarmony through the AltAnalyze graphical user-interface, the user must start the program, select the appropriate species, select the Additional Analyses Menu and then Cell Classification Menu. Pre-compiled graphical user-interface distributions are provided from http://altanalyze.org and the command-line version from GitHub or via installation from PyPI. Details regarding required and optional input files and additional information regarding the use of cellHarmony on a computational cluster are provided in Supplemental Information.

#### Cell-alignment from community clustering

To identify equivalent cell-types or cell-states from two independent scRNA-Seq datasets, cellHarmony employs a community clustering strategy to produce a network graph and define communities in both the reference and query dataset. In short, this approach initially matches communities of cells across samples and then selects the closest matching reference cell for each query cell, where a single reference cell can match to multiple query cells. This function performs the following steps:

1. *Define communities (partitions) within each of the datasets*: Prior to defining communities, each dataset is restricted to the cell-population specific marker genes previously defined for the reference. When using ICGS version 2 unsupervised results (default), these markers are the top 50 cluster-specific marker genes for each cluster. The query and reference files are imported as Cell Ranger (10× Genomics, version 1.0–3.0 supported) sparse matrices (h5 or mtx) or as tabular input files (counts or scaled). By default, cellHarmony uses the ICGS-NMF produced output heatmap tab-delimited text file as its reference with log2 barcode normalized counts data (Supplemental Information – Input Data). Utilization of each of these inputs produces equivalent downstream results. From this sparse matrix data, the program identifies the *k*-nearest neighbors (*k* =10 by default) for each cell using the python package Annoy (18) and creates a graph of these neighborhoods using the networkx python package. Once created, Louvain clustering is performed with the lowest possible resolution to find maximal partitions (*r* = 0). We denote the resulting communities of reference sample cells by {Cr_1_, Cr_2_, …, Cr_s_} and {Cq_1_, Cq_2_, …, Cq_t_} for the query sample communities.
2. *Find the closest matching communities between the query and reference*: For each community in the reference sample (Cr_i_), a centroid mCr_i_ is calculated from the constituent cells using a simple mean for each gene and likewise for each query sample community (mCq_i_). All pairwise similarities *r_ij_* (numpy corrcoef function) between query (mCq_*i*_) and reference (mCr_*i*_) community centroids are computed and the most highly correlated (Pearson coefficient) reference community for each query community is identified. We represent the result of this matching process for query communities as {Cq_*j*_:Cr_*i*_; ∀_*j*_, *i*:max(*r*_*ij*_)}.
3. *Find the closest matching reference cell for each query cell and propagate the label*: The gene expression of each cell within the query community of a pairing {Cq_*j*_:Cr_*i*_} is compared (Pearson correlation coefficient) to each cell in the matched reference community Cr_*i*_ to find the closest match. The original cluster label (i.e. user-supplied label or ICGS/Seurat cluster number) assigned to the closest matching reference cell is then projected to the query cell. A new heatmap is created wherein every query cell is placed adjacent to its closest matching reference cell, reference cells being grouped by the original clustering (see Input Data). However, query cells with a Pearson alignment correlation coefficient lower than the user defined threshold (default = 0.4) are excluded from downstream combined data visualizations, differential expression, and system level prediction analyses. The result is a set of aligned cell populations wherein each cell population consists of all reference cells with a common label and any query cells with that label. We denote the set of aligned cell populations by {*A_k_*, *k* = 1, …, *K*} where *K* is the number of ‘original’ clusters in the reference dataset. The relative frequency distribution of the (aligned) populations within the reference and query samples is compared using a series of *K* Fisher's exact tests. Alternative options for running these functions and performance are described in the Supplemental Information.

#### Differential expression analysis

To identify impacted genes, networks, and cellular processes across cell-populations, cellHarmony performs a differential expression (DE) analysis between each aligned query and reference cell population {*A_k_*, *k* = 1, …, *K*}. Differential expression is performed using an empirical Bayes moderated *t*-test with Benjamini–Hochberg (BH) false discovery rate (FDR) correction applied separately within each cell-type population. An overall moderated t-test for each gene comparing the pooled query cells to pooled reference cells is also performed, with the BH correction applied. We denote the adjusted *P*-values from these analyses by {*p*_1_, *p*_2_, …, *p_K_*} and *p*_O_ for the overall test. The default threshold for differential expression is fold >1.5 and *P* < 0.05 (FDR corrected). Users can modify these thresholds within the graphical user interface or from the command-line (Supplemental Information). This process is automated for cell populations in the two compared datasets that share the same provided or assigned labels between the reference and query, respectively. The K pairwise query-to-reference comparisons are performed for cell populations in which at least 20 cells are present from both the query and reference datasets. For datasets with fewer than 200 total cells (i.e. Fluidigm C1), this requirement is relaxed to 4 cells in each aligned cluster. The results are saved to tab-delimited text files with summary statistics and basic annotations, along with summary graphical outputs, and are used for downstream systems-level analyses.

#### Systems-level predictions

In comparisons where a molecular, genetic, or chemical perturbation results in abnormal cell biology, cellHarmony can be used to identify which cell populations are principally impacted in both cell frequency and gene expression. By examining the differences that are shared among different cell clusters and those that are unique to a specific cell clusters, cellHarmony enables the determination of holistic pathway and gene regulatory network impacts. Specifically, cellHarmony determines whether gene expression differences across cell clusters are global (impacted in most cell populations), local (i.e. cell cluster-specific), or co-regulated/regional (i.e. specific to a subset of cell clusters). Note that although some clusters can be excluded from the differential expression analysis due to insufficient cell population size (see above), we continue using the notation for the full set of aligned clusters {*A_k_*: *k* = 1, …, *K*}.

To assess global gene up- and down-regulation, the differential expression analysis is repeated for all query cells compared to all reference cells, regardless of the cluster to which they are aligned. Only genes for which *p*_O_ < 0.05 and *p*_*i*_ < 0.05 for at least 2 {*i*:1, …, *K*}, with consistent direction of fold change, are considered as candidates for the globally up-regulated or globally down-regulated group, depending on the direction of the fold change. Genes that meet the DE criteria stated above *for only one cell population* form the local DE groups (i.e. *p_i_* < 0.05 for only one {*i*:1, …, *K*}) and represent cell cluster-specific profiles. To define additional predominant patterns of co-regulation (common effect among multiple but not all clusters) for each gene, the cell cluster-specific *P*-values comparing each query to reference cluster (*A*_*k*_) are collected into a vector of length *K*. The vector is recoded to entries of 1, –1 or 0 as follows: 1 if *P* < 0.10 & sgn(logfc) = 1, –1 if *P* < 0.10 & sgn(logfc) = –1, 0 otherwise to indicate up-regulation (1), down-regulation (–1), or no significant alteration (0). The *P*-value threshold of 0.10 was chosen to provide increased sensitivity in clusters with small numbers of cells. For example, a gene with pattern [0,1,0,1] would represent co-regulated DE for clusters 2 and 4. The four most frequently occurring patterns of co-regulation (excluding global and cell cluster-specific patterns) are selected for an additional round of differential gene expression analyses, comparing query to reference cells in the resulting aggregated cells clusters. For example, if the top pattern is [1,1,0,0], a test of pooled clusters 1 & 2 in the query will be compared to clusters 1 & 2 in the reference scRNA-Seq dataset, yielding *P*_(co-reg)_. Each gene from the cell cluster-specific comparisons (local), is then assigned to the specific comparison group (global, local, or co-regulated) in which they are most altered, based on the smallest *P*-value of all comparisons, i.e. among {*p_i_*: *i* = 1, …, *k*, *p*_O_, *p*_(co-reg)_}. The final genes are limited to significant genes in the first cell cluster differential analysis, excluding new genes identified in the global or regional analysis, to prevent bias due to cell-type frequency variation in the query and reference (e.g. more B-cells in query versus reference, highlighting B-cell upregulated gene expression).

To produce a unified representation of these differences, which can consist of dozens of differential expression comparisons, cellHarmony creates a combined heatmap with genes assigned to specific comparison groups. The cells in the heatmap are ordered by the original cell populations, with the genes ordered according to global, co-regulated, and local categorizations (p-value ranked), with up-regulated and down-regulated genes displayed as adjacent clusters. A gene set enrichment analysis is performed on the resultant gene clusters with the Pathway Commons database (human and mouse) or Gene Ontology (other species) (19,20). Putative gene regulatory relationships in these gene modules are predicted using a second gene set enrichment analysis with the TRRUST, PAZAR and Amadeus databases combined to identify likely upstream transcriptional regulators and highlight clustered embedded transcription factors (21,22). Finally, these gene regulatory networks are further visualized using the ‘igraph’ pathway library and these same transcription factor target databases for each comparison.

### Statistical methods for comparative analyses

To evaluate the agreement in label assignments for different label projection methods, we used the Adjusted Rand Index (ARI) as proposed by Hubert and Arabie (23) using the R package ‘clues’ (24). Accuracy is assessed as the proportion of correct assigned labels for a given alignment algorithm, where perfect accuracy is defined as matching aligned and author assigned labels. To evaluate methods for differential expression analysis, we used type I error probability and false discovery rate (FDR), each assessed at a nominal error rate of 0.05 (5%), and statistical power (see Supplemental Information for definitions).

### Evaluation datasets

See the Supplemental Information for details regarding the software inputs and outputs, heart scRNA-Seq produced in these studies, external validation datasets used, external software evaluation, data processing and analysis parameters, along with user guidelines for cellHarmony parameter tuning.

## RESULTS

The cellHarmony pipeline was created to rapidly and accurately align comparable scRNA-Seq datasets to identify the global, regional, and local molecular impacts of diverse perturbations. For this purpose, cellHarmony uses a graph-based strategy to produce disjoint networks from a clustered single or combined reference against an un-clustered query (Figure 1A and B). Alignment through community clustering provides three important advantages in the alignment of different scRNA-Seq datasets. First, individual cells can be quickly matched between large scRNA-Seq datasets by finding the most similar communities between datasets prior to matching individual cells in those partitions. Second, the produced alignments will be more stable than individual cell alignments which are inherently biased towards outlier cells. Finally, the alignment to cell centroids is highly reliant on the final cluster definitions, in which sub-clusters may be present or clusters inappropriately aggregated. Cell populations and cluster labels (e.g. cell-type names optionally provided by the user) in the reference can be automatically assigned using the embedded unsupervised algorithm Iterative Clustering and Guide-gene Selection (ICGS) (17), which has been consistently shown to identify transitional cell states and exceedingly rare cell types from large scRNA-Seq datasets (ICGS-NMF output) (1,17,25–29). While reference cell alignment is an important independent objective in many studies (i.e. developmental ordering of cells, cell-type identification), cellHarmony's primary innovation is the creation of systems-level models of cellular and transcriptional differences in discrete and co-regulated cell populations. The result is a global view of molecular perturbations across cell types to determine which cell-populations, associated pathways, and transcriptional networks are impacted. To achieve this aim, the software implements a unique comparison and aggregation approach to identify impacted gene and regulatory programs that may be restricted to individual cell clusters, shared more broadly between specific cell clusters, or that are common to all populations (Figure 1C–F). Visualization of these organized gene modules allows for the determinations of which pathways and gene networks are playing important roles in which cell populations.

### Label projection performance

**Figure 2.**
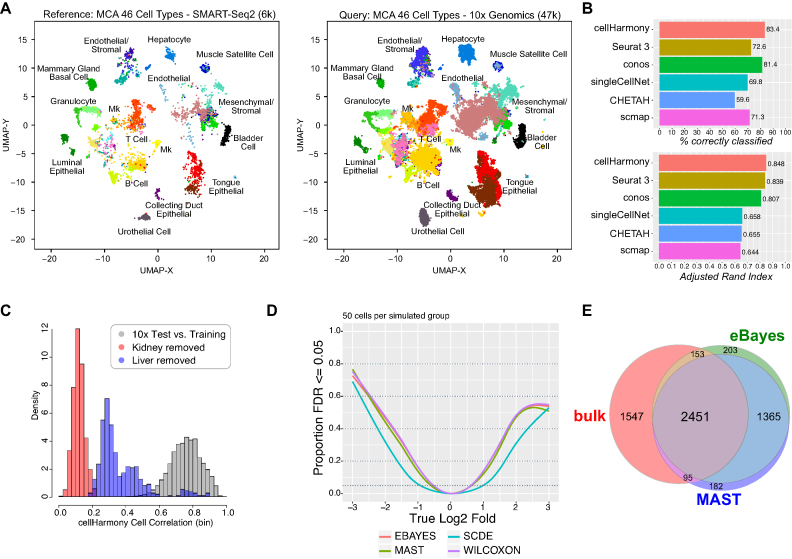
cellHarmony produces accurate alignments across technologies. (**A**) cellHarmony produced UMAP projection of cells from 12 Tabula Muris tissues profiled using two different single-cell methods, SMART-Seq2 (reference, left) and 10x Genomics (query, right). 10x Genomics cell population labels were transferred from the SMART-Seq2 through community-alignment. Cells were restricted in both sets to those with common assigned Cell Ontology annotations by the study authors to allow for evaluation of the alignment agreements. (**B**) Comparison of the cell-label assignment by six independent algorithms (cellHarmony, Seurat3 reference label transfer, conos label projection, scmap, singleCellNet and CHETAH) using the cells and labels from panel C. Results from cellHarmony are reported using variable genes derived from an unsupervised analysis of all SMARTSeq2 cells (ICGS version 2), whereas marker genes for all other applications were derived within those tools. Adjusted Rand Index is reported as the overall measure of agreement to the author provided Cell Ontology labels with confidence intervals for ARI computed using the normal approximation. (**C**) Evaluation of excluded cell types by cellHarmony in the 10× Genomics MCA dataset. The histogram indicates the cellHarmony cell similarity values (Pearson coefficient coefficient) in: (i) 50% of the MCA 10× Genomics cells against the remainder for all 46 cell types, (ii) liver cell hepatocytes aligned against 50% of the MCA 10x Genomics cells (excluding these cells form the reference), (iii) kidney collecting duct cells aligned against 50% of the MCA 10x Genomics cells (excluding these cells from the reference). Both kidney and liver cells should not have an analogous reference cells in the reference set, hence, alignments should be poor. (D, E) Evaluation of differential gene expression sensitivity using the empirical Bayes method. (**D**) Statistical power curves for simulated RNA-Seq data indicate the proportion of tests (features) with FDR-adjusted p-values ≤ 0.05 over a range of log2 fold-change values. Simulated data was generated using the R package ‘splatter’ with 50 cells for each of the two groups (composite of 5 simulated datasets). (**E**) Overlap of differentially expressed genes (DEGs) from T-cells versus B-cells profiled either by bulk RNA-Seq (benchmark data) or scRNA-Seq predicted using the MAST testing procedure or empirical Bayes t-test (eBayes) (see Supplemental Information).

To specifically evaluate the performance of cellHarmony relative to alternative label projection methods, we applied cellHarmony, conos, Seurat3, scmap, CHETAH and SingleCellNet to a large mouse cell atlas (MCA) dataset (Tabula Muris) with previously defined Cell Ontology classifications (n=46). This dataset includes 12 matched tissues profiled using two complementary scRNA-Seq technologies (10x Genomics and SMART-Seq2) and capture methods (unbiased versus Flow Cytometry). As such, cell-type definitions are considered highly rigorous, although additional undefined cell clusters are expected. It should be noted that another label projection method, Garnett, could not be evaluated as it requires unique genes for all evaluated cell populations, which is not the case for the 46 MCA cell-types (i.e. B-cells from different tissues) (Supplemental Information). Similarly, the algorithms RCA (30) and MNN (31) do not explicitly perform label projection and hence were also excluded (see Table 1, Supplemental Information). Although the two scRNA-Seq datasets generated by the Tabula Muris consortium were produced using independent single-cell methodologies, UMAP and heatmap projections of cellHarmony-aligned datasets indicate highly overlapping cell type projections for the major cell populations (Figure 2A and Supplementary Figure S1A, B). Using the Adjusted Rand Index (ARI) for alignment of the 10× Genomics query to the SMART-Seq2 reference to quantify similarity, community-based alignment in cellHarmony had improved similarity to the author-defined ground truth (ARI = 0.85) with high accuracy (83%), relative to conos (ARI = 0.81, accuracy = 81%), Seurat3 (ARI = 0.84, accuracy = 73%), scmap (ARI = 0.64, accuracy = 71%), CHETAH (ARI = 0.65, accuracy = 60%), and scNet (ARI = 0.66, accuracy = 70%) (Figure 2B). While cellHarmony was evaluated using markers selected from an unsupervised analysis (ICGS version 2), near identical results were obtained using MCA Ontology cell-population marker genes (Supplementary Figure S1C). We find that mis-classifications from cellHarmony could largely be explained by alignment of cells with same or highly related cell-types across tissues (e.g. Muscle Macrophage as Trachea blood cell) (Supplementary Figure S1D). To determine the thresholds at which cellHarmony predictions fail to be accurate, we excluded two tissue-restricted cell populations that clearly separated out from other cell-types in the UMAP graph, liver hepatocyte and kidney collecting duct, and classified those cells back into the filtered reference dataset. These data indicate that cellHarmony is more likely to produce true positive alignments above a Pearson correlation >0.3 (default cutoff >0.4), with alignments below that range indicating likely false positive associations for cells from the same technological platform (Figure 2C). Hence, cellHarmony is accurate across technological platforms to yield high precision cell population alignments for the downstream identification of cell population-specific impacts.

### Cell population comparison of differential expression

To obtain higher order insights, cellHarmony requires an effective method to determine gene expression differences when comparing query cells to reference cell clusters. For such comparisons, it is expected that the end-user identifies any possible batch-effects and corrects for them as needed prior to performing cellHarmony. For differential expression analysis, cellHarmony applies an empirical Bayes (eBayes) moderated t-test method (32). We speculated that this computationally efficient method would be appropriate for scRNA-Seq datasets in which a high degree of missing values is present, as it moderates the standard error towards a common value. To verify the performance of this method with scRNA-Seq, we compared eBayes to three algorithms frequently applied for scRNA-Seq differential expression analysis (MAST, SCDE and Wilcoxon) using simulated data (splatter package) in addition to matched single-cell and bulk RNA-Seq data (Figure 2D and Supplementary Figure S2A, Supplemental Information). Compared to alternative algorithms, we find eBayes can accurately identify cell-type-specific differences in gene expression in scRNA-Seq data with statistical power comparable to state-of-the art algorithms (e.g. MAST), while providing Type I and FDR error control at a nominal 5% levels (mean observed Type I error rate across the five replicates of 0.039 and 0.44 for 50 and 100 cells per group, respectively, and likewise 0.025 and 0.034 for FDR). Application of eBayes to scRNA-Seq with matched bulk RNA-Seq (B-cells versus T-cells), identified a nearly identical proportion of bulk RNA-Seq verified differentially expressed genes as MAST and SCDE (Figure 2E and Supplementary Figure S2B).

### Using cellHarmony to identify disease gene networks

To evaluate the ability of cellHarmony to discover known and novel cell-population impacts, we applied this tool to four scRNA-Seq collections, spanning three technologies (Fluidigm, DropSeq, 10× Genomics) and two diseases (myocardial infarction and Acute Myeloid Leukemia). For both diseases, we selected datasets with comparable bulk RNA-Seq data as a means to validate the observed gene expression differences. Notably, many well-defined drug targets and key driver gene expression changes are already known for both diseases.

**Figure 3.**
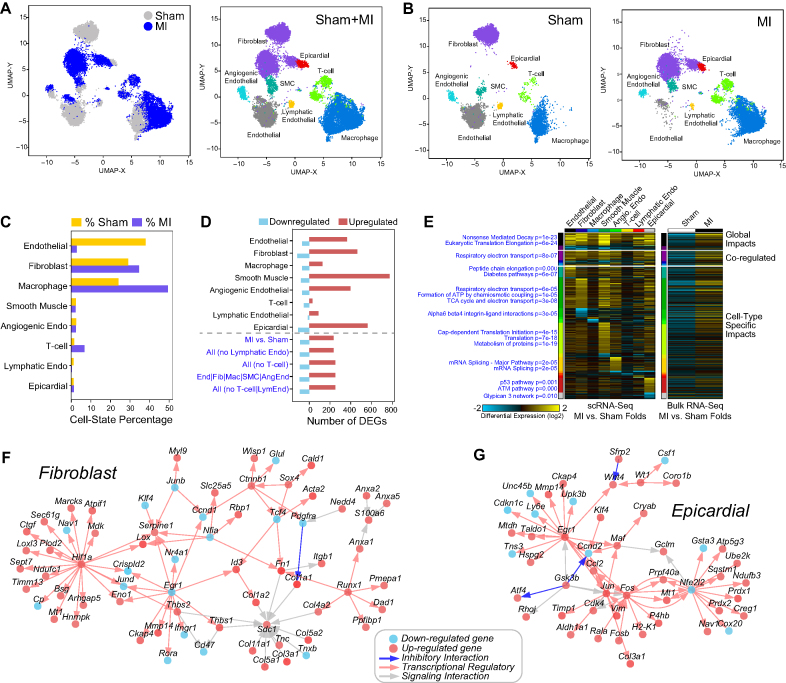
Identification of cell-type specific regulatory networks in cardiac ischemia. (**A**) cellHarmony UMAP projection of mouse heart scRNA-Seq, comparing myocardial infarction (MI) to non-diseased hearts (Sham surgery). Cell cluster labels and colors were assigned by cellHarmony (MI) or from the Seurat analysis (Sham). (**B**) Separation of the combined UMAP projections for reference Seurat Sham clusters and aligned MI clusters. Differences in the projected population locations in the MI are attributed to disease-associated transcriptomic changes. (**C**) Cell population percentages for cells from Sham and MI. (**D**) Number of differentially expressed genes for each cell cluster (local), global (all AML cells versus all wild-type) and regional (coregulated cell clusters) comparisons for MI versus Sham (fold>1.5, empirical Bayes moderated *t*-test *P* < 0.05 (FDR adjusted)). (**E**) Ordered gene expression changes (upregulation and downregulation) for each global, regional and the cell population-specific comparison, based on relative gene expression *P*-value rankings, along with associated statistically enriched pathways and enrichment *P*-values (blue text) (left panel). The same genes are shown in the right panel for comparable bulk RNA-Seq of MI versus Sham, from a prior study (GSE96561). (**F**, **G**) cellHarmony produced gene network displaying putative regulatory interactions (red arrows) among up-regulated (red notes) and down-regulated genes (blue nodes), separately for fibroblast (F) and epicardial (G) MI versus Sham comparisons.

Myocardial infarction (MI), commonly known as a heart attack, is the leading cause of death in developed countries. While decades of transcriptomic analyses have revealed the global impact on cardiac tissue, little is known about the specific impacts to individual cell populations. We performed Drop-Seq on a mouse model of MI and age-matched controls with sham surgery. MI is known to result in large global gene expression changes associated with cell death, cellular infiltration, and injury induced cellular remodeling that make its comparison to controls complex (33). Applying cellHarmony to Seurat clustering of the Sham references finds that, while all cell populations present in the healthy heart are present in the diseased, MI results in gene expression changes that shift the location of cell populations within the combined UMAP graph (Figure 3A and B). Specifically, visualization of the aligned MI cells within the cellHarmony heatmap shows that fibroblasts adopts a weak combined smooth muscle, epicardial, and endothelial gene expression program (Supplementary Figure S3A). Further, MI results in a loss of endothelial cells and gain in infiltrating macrophages (Figure 3C). It should be noted that joint-alignment using Seurat (version 3) dataset integration finds cell clusters that are restricted to Sham (endothelial) or MI (fibroblasts) that represent distinct cell populations (Supplementary Figure S3B and C). While biologically informative, the occurrence of such separate clusters hinders the direct-automated comparison of these populations between MI and Sham.

In MI, cellHarmony gene expression differences across all cell populations indicate consistent upregulation within each specific and clustered cell population (Figure 3D). While little gene downregulation is denoted in the combined heatmap representation of this data, we find regulation of diverse pathways with increased cell population-specific gene expression. These changes are broadly reflected in comparable bulk RNA-Seq data from an independent study (33), indicating that differential expression is likely valid rather than a technical artifact (Figure 3E). Moreover, examination of the predicted transcriptional regulatory networks for each specific cell population comparison identifies well defined MI-associated gene expression differences in human Fibroblasts (up-regulation of *Col1a1, Col1a2, Col3a1, Col4a2, Col5a1, Col5a2* [Collagen scar] *Fn1, Thbs1, Mmp14*) along with novel implicated regulators which agree with prior literature (Figure 3F) (34–37). While the two central transcriptional nodes in this network, *Hif1a* and *Runx1*, have been previously implicated in MI and even as possible targets for therapy, these markers have not been explicitly associated with the MI fibroblasts (38–40). While another central node in this fibroblast network, *Egr1* was down-regulated, we note that in the Epicardial network, *Egr1* was also central but up-regulated, in combination with other immediate-early central node genes *Jun* and *Fos* (Figure 3G). This observation is important, as *Egr1* is a well described transcriptional regulator and target for therapy in MI and is likely playing very different roles in these two different cell types (41–43). Hence, automated cellHarmony analysis is able to identify the likely cell populations that underlie therapeutic responses and well-defined cell-type specific mediators of disease.

### Identifying clonal cell origins in cancer

Cancers of the blood, such AML, can derive from diverse progenitor populations to gain self-renewal potential. While AML results from myeloid progenitors, genomic variants and disease subtypes are likely to derive from distinct stem or progenitor populations, most of which remain largely unknown. The comparison of scRNA-Seq datasets provides a means to determine which cancer clones associate with known healthy progenitor cell types. To understand the diversity of such cancer cell origins and response to therapy, we applied cellHarmony to three AML scRNA-Seq collections: (i) a murine model of cytogenetically normal (CN) AML, (ii) an erythro-leukemia patient with patient matched control and (iii) a time-course of AML therapy.

**Figure 4.**
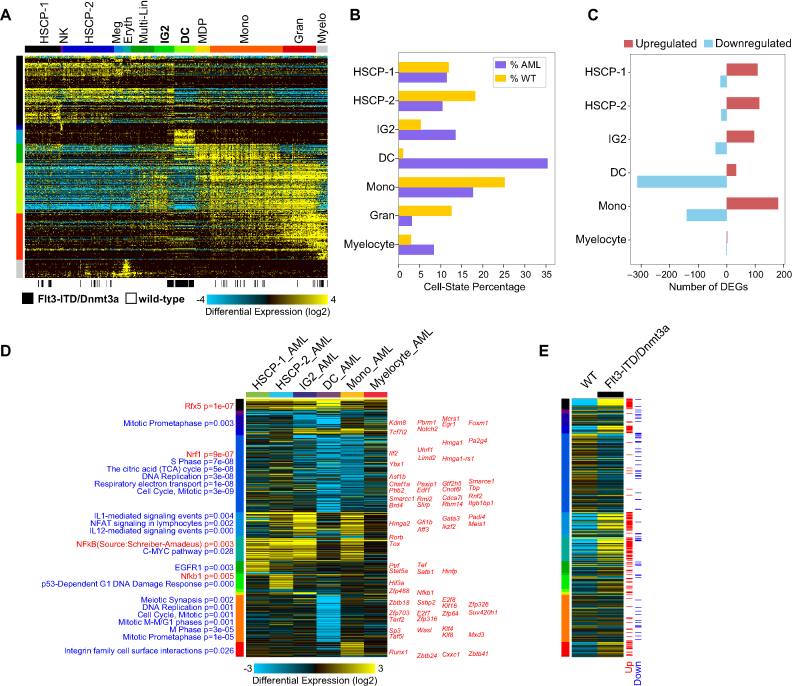
Cell population-specific impacts in transitioning progenitors and AML. (**A**) cellHarmony alignment of previously profiled splenic c-kit-positive AML cells relative to ICGS results from all captured normal mouse bone marrow hematopoietic progenitors (reference sample, Olsson *et al.* 2016). (**B**) AML cells were aligned most frequently to annotated dendritic cells (DC), monocyte progenitors (Mono), and previously described bipotential hematopoietic progenitors (IG2, Olsson *et al.* 2016). The AML DCs were high enriched relative to wild-type DCs by cellHarmony Fisher Exact test (*P* = 4.8E–24). C) Number of differentially expressed genes (DEG) by cell cluster (upregulated and down-regulated) for AML versus wild-type cell populations (fold > 2, *P* < 0.05, FDR adjusted). Negative numbers on the x-axis indicate downregulation (i.e. ‘-100’ means ‘100 downregulated genes’). (**D**) Heatmap of AML versus wild-type fold changes for all significant differentially regulated genes from panel C. Genes are grouped as global, regional, and local transcriptomic differences (DEG p-value ranked), with DEGs demonstrating global regulation shown at the top of the heatmap, co-regulated cluster impacts in the middle, and cell population-specific impacts at the bottom. The regional or co-regulated clusters indicate shared patterns of gene expression across multiple cell populations (e.g. HSCP-1 + HSCP-2 + IG2 + Mono). For each pattern, a separate up-regulated and down-regulated cluster is shown. Known transcription factors are displayed to the right of the heatmap where present by cellHarmony and enriched Pathway Commons gene-sets (blue) and transcription factor target sets (red) are displayed on the left next to each cluster in ranked order of significance (bottom to top). (**E**) Corresponding gene expression differences from bulk RNA-Seq of the AML model compared to wild-type bone marrow controls as an independent verification. Red lines = upregulated genes (fold >1.5, empirical Bayes moderated *t*-test *P* < 0.05 (FDR adjusted)), Blue lines = downregulated genes (fold < –1.5, empirical Bayes moderated *t*-test *P* < 0.05 (FDR adjusted)).

In the murine model of CN-AML, mice carrying both Flt3-ITD and Dnmt3a mutations (44) were compared to healthy bone marrow hematopoietic progenitors (BM) (1) which include well-defined and experimentally validated transition cell states. Here, cellHarmony primarily aligned AML cells to wild-type dendritic cells, monocyte progenitors and a newly described population of bi-potential monocytic or granulocytic progenitors (IG2) cells (Figure 4A and B). As IG2s are considered short-lived cellular intermediates, their role in both normal and abnormal hematopoiesis is unknown. While hundreds of genes were differentially expressed between matched AML and healthy BM, the cellHarmony integrated visualization indicates that these genes largely segregate those shared among discrete lineage transitions, including those with apparent monocytic restricted potential, and those specifically impacted in distinct cell types (e.g., dendritic cells), with specific pathway and gene-regulatory impacts (Figure 4C and D). For example, genes consistently up-regulated in the transition to monocytes are characterized by activation of C-MYC and NF-kB, whereas down-regulated genes in both monocyte and dendritic cell progenitors show an exit from cell-cycle. This analysis further highlights hematopoietic stem progenitor (HSCP) programs (*Gfi1b, Meis1, Gata3, Ikzf2*) are active in later progenitor populations (AML IG2 and monocytic), where they should normally be down-regulated (1). These genes were previously shown to be aberrantly expressed in human AML (45–48). Notably these cell type-specific differences were reflected in in bulk RNA-Seq from these same cells (Figure 4E).

**Figure 5.**
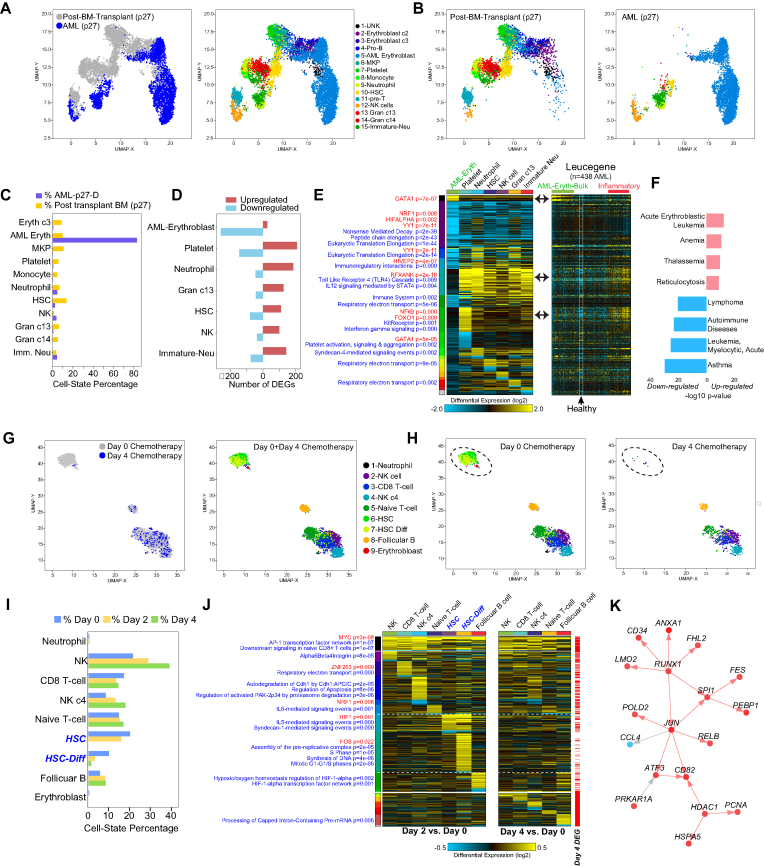
Assessing temporal changes in cell population-specific gene regulatory programs in Acute Myeloid Leukemia. (**A**) UMAP projection of ICGS-NMF scRNA-Seq clusters from bone marrow mononuclear cells from a single-patient (AML027) after bone marrow transplantation and a cellHarmony aligned diagnosis AML sample. (**B**) Separation of the combined UMAP projections for reference post-transplantation clusters and aligned AML clusters. (**C**) Cell population percentages for cells from post-transplantation and AML. The diagnosis AML erythroblast cluster represents the dominant cell population in the AML patient. (**D**) The number of corresponding gene expression changes in the AML compared to the post-transplantation. The AML erythroblast cluster is characterized by downregulation while all other clusters by upregulation. (**E**) Ordered gene expression changes (upregulation and downregulation) for each global, regional and the cell cluster-specific comparison, with transcription factor target gene-sets displayed on the left (red) and pathways (blue) to the left of the heatmap with corresponding enrichment p-value (left panel). The same genes are shown in the right panel for comparable bulk RNA-Seq from the Leucegene AML RNA-Seq cohort with 438 patients and 16 donor CD34+CD45RA- cord-blood samples (row median normalized). Samples indicated as AML Eryth are those with a matching GATA1 transcription factor target enrichment in the left panel heatmap and those denoted as inflammatory correspond to gene clusters with an enrichment in interferon-gamma and TLR gene set enrichments. (**F**) Disease Ontology gene-set enrichment with the program ToppGene of the two Leucegene patient groups high GATA1 and low inflammatory gene expression versus the converse sample sets. Red bars indicate statistical enrichment of upregulated (GATA1 high) gene sets and blue, downregulation associated genes (inflammatory). (**G**, **H**) Analogous plots to those shown in panels A and B, respectively, for two scRNA-Seq timepoints of a single-patient's blood at Day 0 and Day 4 of chemotherapy. (**I**) Cell population percentages for Day 2 and Day 4 cells aligned to Day 0 ICGS-NMF clusters. No cells were aligned for HSC cells at Day 4 and only 11 cells for HSC-Differentiating at Day 4 (too few for differential expression analysis of those clusters). (**J**) Ordered AML time-course heatmap, in which ordered genes for both Day 2 and Day 4 versus Day 0 were combined. Differentials for Day 2 are shown on the left panel and Day 4 on the right, with red tick marks denoting genes significantly differentially expressed in Day 4 versus Day 0. (**K**) The cellHarmony gene network for the differentiating HSC cluster at Day 2 versus Day 0.

To see whether cellHarmony is effective at identifying cell population-level genomic differences relevant in diseased patient samples, we analyzed human leukemia scRNA-Seq datasets before, during, or following therapy. To minimize potential batch and donor effects for differential analyses, we selected scRNA-Seq from a pre-transplantation leukemia bone marrow biopsy relative to a post-transplantation biopsy on the same patient, although the donor genetics will differ from the recipient (49). Alignment of the diagnostic query to the post-transplantation sample reference found an expected decrease in cellular diversity in the leukemia diagnostic sample and significant expansion of erythroblast compartment in the leukemia by UMAP visualization (Figure 5A–C and Supplementary Figure S4A). Consistent with this observation, this patient was diagnosed with erythro-leukemia, which is characterized by proliferation of erythroblastic precursors (49). Interestingly, the most divergent gene expression differences in pre- versus post-transplant are found in Erythroblasts, which were characterized predominantly by gene down-regulation, where as other cell-types were principally characterized by up-regulation (Figure 5D and E). To determine whether such signatures were diagnostic for leukemia type, we analyzed bulk-RNA-Seq in a large un-annotated adult AML cohort consisting of 438 patients (Leucegene) and 16 donor CD34+CD45RA- cord-blood samples for the same gene modules (50). Comparison of patients with the high-GATA1 signature (and low inflammatory) to the high inflammatory (and low-GATA1) yields genes with expected enrichment in previously described erythro-leukemia and inflammatory/autoimmune disorder disease ontology terms, respectively, providing evidence that these signatures are indeed diagnostic (Figure 5F).

As a final evaluation, we considered a recent AML treatment scRNA-Seq time-course (Days 0, 2 and 4) with a combinatorial drug therapy to induce durable remission. The study authors use this data to demonstrate a loss in blood leukemic stem cell populations during therapy and metabolic disruption. Similarly, cellHarmony automatically indicates the same gradual loss in stem cell blood populations during treatment and enriched gene expression changes in analogous metabolic pathways (Figure 5G–I, Supplementary Figure S4B, Supplementary Table S1). However, our analysis also implicates time and cell-type dependent transcriptional regulatory networks, which suggest that the major population gene expression impacts happen at Day 2 of therapy (global and cell population specific up-regulation) (Figure 5J). Among the gene expression differences most pronounced at Day 2 were those restricted to the two Leukemia stem cell-like populations. Examination of these changes reveals dozens of highly significant impacted pathways, notably syndecan-1, IL5, TGF-beta, EGFR, IFN-gamma, IGF-1, ErbB1, integrin and mTOR mediated signaling pathways (Supplementary Table S1). These impacted pathways have well-described regulatory roles in proliferative signaling, which is enriched among up-regulated genes (Mitotic G1-G1/S phases) in concert (Figure 5J). These differences were further exemplified by the cellHarmony predicted gene-regulatory network for differentiating HSCs, which highlight a core stem cell and proliferative regulatory program orchestrated by up-regulation of *RUNX1*, *JUN* and *HDAC1* (Figure 5K). Such transcriptional differences represent new potential biomarkers for intermediate response to therapy as well as novel possible therapeutic targets.

## DISCUSSION

Single-cell RNA-Seq continues to enable exciting insights from healthy and diseased tissues. Standardized and efficient workflows to explore such diversity benefit the research community by highlighting global and cell population-specific programs that can otherwise remain hidden. cellHarmony provides such reproducibility through the fast alignment of independent scRNA-Seq graphs using a community alignment approach. As such, the approach remains scalable to large datasets, due to its low memory footprint, and will be less sensitive to outlier effects through the use of neighborhood matching. To maximize the use of this application, both a stand-alone python implementation of this tool is provided along with an integrated workflow with a graphical user-interface. While this method can accurately align diverse cells from different tissues and across different single-cell technologies, its principle power is in the determination of global, regional, and local differences among cell clusters at the transcriptional, pathway, and regulatory-network level. As demonstrated, such insights include the localization of critical disease transcriptional changes to specific cell types, improved understanding of the specificity of drug targets to specific cell types in disease, improved diagnostic biomarkers, and novel regulatory and signaling networks that can inform therapy. Importantly, we are unaware of any other scRNA-Seq pipelines that can perform these integrated tasks (see Table 1).

While cellHarmony is able to yield exciting new insights from disease scRNA-Seq datasets, a number of important challenges still remain. These challenges include the comparison of large single-cell patient cohorts, with variable cell numbers per sample, confounded by batch, genetics, and other effects and the explicit integrated analysis of multi-time-point datasets. Further, cellHarmony reported gene expression differences will inherently be less confident when comparing aligned clusters with highly varying sizes (e.g. dozens of cells versus thousands). Likewise, there is always the chance that similar, but not exact cell types will be inappropriately labeled in different datasets. As such, this approach should be considered along with those for joint-alignment to identify additional cellular granularity not clear from the reference alone. Although new specialized and integrated approaches are needed to address these goals, our approach provides an important starting framework for more complex study designs involving scRNA-Seq through community alignment. With increasing use and decreasing expense of scRNA-Seq technologies, we anticipate approaches such as cellHarmony to become necessary to derive higher order insights into the investigation of pharmacological and disease heterogeneity.

## DATA AVAILABILITY

All reported datasets used in this paper are publicly available with the associated accession numbers provided below. Tabula Muris mouse cell atlas scRNA-Seq data (Figure 2) was obtained from GSE109774; mouse bone marrow scRNA-Seq from GSE70245 and GSE77849; myocardial infarction and sham surgery mouse scRNA-Seq deposited to GSE136088; human Acute Myeloid Leukemia (AML) datasets from the 10x Genomics website (https://support.10xgenomics.com/single-cell-gene-expression/datasets/1.1.0/aml027_pre_transplant, https://support.10xgenomics.com/single-cell-gene-expression/datasets/1.1.0/aml027_post_transplant); AML scRNA-Seq time-course from GSE116481; human bulk AML RNA-Seq data (GSE49642, GSE52656, GSE62190, GSE67040) and control cord-blood samples (GSE48846); Human HEK293T scRNA-Seq from the 10X Genomics website (http://support.10xgenomics.com/single-cell-gene-expression/datasets/2.1.0/hgmm_12k); bulk RNA-Seq from T-cells and B-cells (GSE51984); single-cell RNA-Seq from human peripheral blood mononuclear cells from the 10x Genomics website (https://support.10xgenomics.com/single-cell-vdj/datasets). cellHarmony and AltAnalyze are open-source and provided in Github according to the Apache License 2.0.

## Supplementary Material

gkz789_Supplemental_Files
