# Supplementary material for: cellHarmony: cell-level matching and holistic comparison of single-cell transcriptomes
Source: Nucleic Acids Res. 2019 Sep 16;47(21):e138. doi: 10.1093/nar/gkz789 (PMC6868361; doi:10.1093/nar/gkz789)
Supplement: gkz789_Supplemental_Files [file gkz789_supplemental_files.zip › Supplemental.Information.pdf]

# Supplemental Information

## cellHarmony: Cell-level matching and holistic comparison of single-cell transcriptomes

Erica AK DePasquale[1,2,¶], Daniel Schnell[2,3,¶], Phillip Dexheimer[1,2], Kyle Ferchen[4,5], Stuart Hay[2], Kashish Chetal[2], Íñigo Valiente-Alandí[3], Burns C. Blaxall[3,7], H. Leighton Grimes[4,5,6,7,*], Nathan Salomonis[1,2,7,*]
[1] Department of Biomedical Informatics, University of Cincinnati, Cincinnati, OH
[2] Division of Biomedical Informatics, Cincinnati Children's Hospital Medical Center, Cincinnati, OH
[3] Heart Institute and Center for Translational Fibrosis Research, Cincinnati Children's Hospital Medical Center, Cincinnati, OH
[4] Department of Cancer Biology, University of Cincinnati, Cincinnati, OH
[5] Division of Immunobiology and Center for Systems Immunology, Cincinnati Children's Hospital Medical Center, Cincinnati, Ohio, USA.
[6] Division of Experimental Hematology and Cancer Biology, Cincinnati Children's Hospital Medical Center, Cincinnati, OH
[7] Department of Pediatrics, University of Cincinnati School of Medicine, Cincinnati, Ohio, USA.
¶co-first Authors
*co-corresponding Authors

# Contents


**1 Extended Methods for cellHarmony ..... 3**

1.1 User and Command-Line Interfaces ..... 3

1.2 Running cellHarmony on a Cluster ..... 4

1.3 Input Data Files ..... 5

1.4 Combining ICGS Datasets ..... 6

1.5 Programmatic Options and Parameter Tuning ..... 6

1.6 Output Files ..... 7

1.7 Evaluation Datasets ..... 8

1.8 Single-Cell RNA-Sequencing ..... 10

1.9 Evaluation Details for cellHarmony ..... 11

**2 Supplementary Figures ..... 15**

2.1 Supplementary Figure S1 ..... 15

2.2 Supplementary Figure S2 ..... 17

2.3 Supplementary Figure S3 ..... 18

2.4 Supplementary Figure S4 ..... 20

**3 References ..... 21**

# 1 Extended Methods for cellHarmony

## 1.1 User and Command-Line Interfaces

cellHarmony can be run through the AltAnalyze graphical user interface or through the command-line. AltAnalyze is available as pre-compiled binaries (http://www.altanalyze.org) or as source code (PyPI python 2.7 installation or https://github.com/nsalomonis/altanalyze). Both command-line and pre-compiled binaries can be run on the command-line (https://github.com/nsalomonis/altanalyze/wiki/CommandLineMode). The cellHarmony-Align code can also be independently run as an alternative to AltAnalyze (https://github.com/AltAnalyze/cellHarmony-Align). This code-base only performs the community-alignment function without the graphical outputs (heatmap, UMAP, networks) or differential expression. Example files are included in the folder sample_data of this repository for which to base text file inputs (e.g., labels). Labels can be derived from the cluster gene-set enrichment results from ICGS (see sample_data/labels). The cellHarmony-Align library (aka rapid-mode) can be run as:

```
$ python scr/cellHarmony_align.py reference/GSM3489185_Donor_02_h5.h5
query/GSM3489183_IPF_01_h5.h5 alignments.txt --genes genes/markers.txt --label
labels/CustomLabels.txt
```

Where "alignments.txt" is an example of an output file path. When run through AltAnalyze the user must first install a species database. From the command-line, for example:

```
$ python AltAnalyze.py --species Hs --update Official --version EnsMart72 --additional
all
```

The user may alternatively choose to install these databases from the graphical user interface from the main menu (Add New Species button) or when prompted when first started (https://altanalyze.readthedocs.io/en/latest/RunningAltAnalyze/). To open the cellHarmony user interface, start AltAnalyze, proceed to the main menu, select the appropriate downloaded species option, proceed to the Additional Analyses menu and the Cell Classification menu to begin the analysis. Prior to running cellHarmony, the user is advised to have: 1) an ICGS-NMF output folder directory or Seurat results (Reference File), 2) the path of the full expression file corresponding to the reference (dense or sparse matrix formats), 3) labels for each cell in the dataset with cell-type notations (recommended), 4) the path to the full expression file corresponding to the query sample to align to the reference (dense or sparse matrix formats). A description of the input files for analysis are described in the following section.

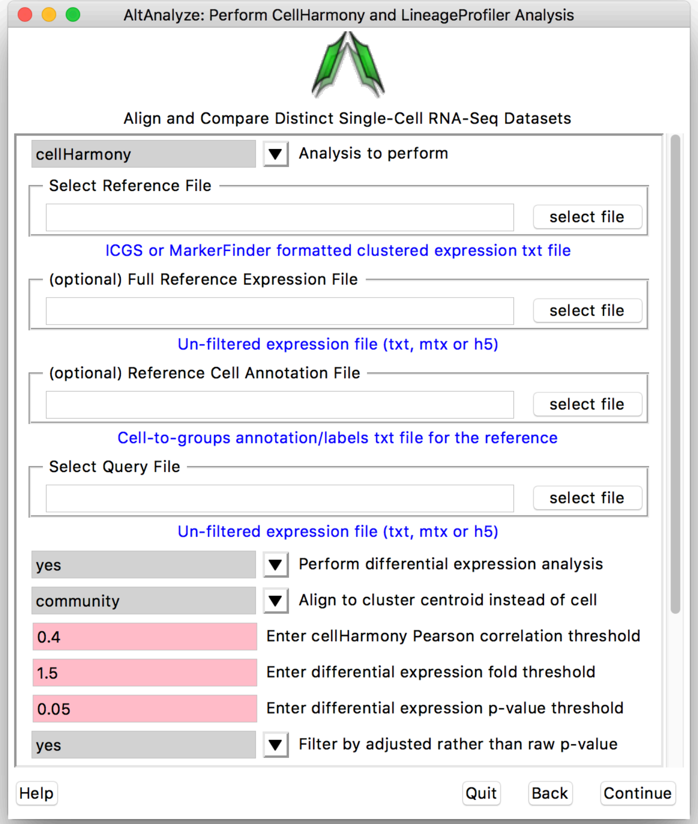


For the command-line interface, cellHarmony can be run as a single command:

```
$ python AltAnalyze.py --cellHarmony yes --input queryFolder/AML.txt --reference
referenceFolder/ICGS-NMF/FinalMarkerHeatmap.txt --platform RNASeq --species Mm --
correlationCutoff 0.4 --referenceType community --performDiffExp True --cellHarmony
yes --adjp True --fold 1.5 --label /labels/BoneMarrow_cluster_names.txt
```

When only performing alignment:

```
$ python AltAnalyze.py --cellHarmony yes --input queryFolder/AML.h5 --reference
referenceFolder/wild-type.h5 --platform RNASeq --species Mm --correlationCutoff 0.4 --
referenceType community --performDiffExp False --cellHarmony yes --label
/labels/BoneMarrow_cluster_names.txt --genes markerGeneFolder/markerGenes.txt
```

## 1.2 Running cellHarmony on a Cluster

We have observed that cellHarmony may produce an error when run on a cluster, specifically when nodes without Advanced Vector Extensions (AVX or AVX2) are selected. This is due to a requirement of the community clustering python libraries. To avoid this issue, request a node with AVX. For example, using bsub: bsub -M8000 -W 4:00 -J testname -n 4 -R "span[hosts=1]" **-R avx,avx2** -o log.out

## 1.3 Input Data Files

cellHarmony can be run in three major modes: 1) “rapid-mode” of two scRNA-Seq datasets, 2) alignment and visualization with no differential expression, and 3) full analysis workflow. Options 2 and 3 require an AltAnalyze format heatmap text file for data visualization and marker gene selection. This heatmap includes the output of unsupervised population detection with ICGS version 2 (ICGS guide-gene folder, ICGS-NMF markers folder or DataPlots/Marker finder output or other produced heatmap). Instructions for creating this file from the output of Seurat can be found on the cellHarmony website (http://www.altanalyze.org/cellHarmony/). When only aligning two scRNA-Seq files (rapid-mode), users must supply the following input:

- (File option 1) Two sparse-matrix (h5 or mtx format) scRNA-Seq dataset files (query and reference). For mtx files (Cell Ranger, version 1-3), the mtx directory must contain a barcodes.tsv, genes.tsv, or features.tsv file. cellHarmony currently supports only h5 or mtx files from the same version of Cell Ranger.
- (File option 2) Two tab-delimited text files with log2 read-normalized expression data (query and reference). These files can contain all genes or be restricted only contain marker genes if desired. The file must contain a gene column (column 1) and a barcode row (row 1).
- Tab-delimited marker genes text file. Genes in column 1.
- (Optional) A tab-delimited label file, with cell barcodes/IDs (column 1) and labels (column 2).

The output of the rapid-mode analysis is a tab-delimited text file with: A) the query and aligned reference cell barcodes, B) aligned communities (cell partitions) and C) user-supplied reference labels (optional). In addition to these same input files, to obtain the other standard outputs of cellHarmony, users must additionally supply:

- An ICGS results directory heatmap file (see above directory locations) along with an ExpressionInput folder in the same parent directory containing the full gene normalized expression file (log2 or non-log). For Seurat, users must save the equivalent format files in the same folder structure. A script is provided at: http://www.altanalyze.org/cellHarmony/ to produce this output from R. This heatmap text file should be selected as the cellHarmony reference.

The query file can be a tab-delimited or sparse matrix input. If sparse, this file will be exported to a dense format, column normalized log2 file to the same directory as the h5 or mtx file (options 2 and 3). This file

will be used for joint visualization (heatmap, UMAP) of the query and reference and downstream differential expression, network, pathway, and systems analyses.

Batch effects should be evaluated, and if necessary, corrected prior to cellHarmony alignment using external tools (e.g., MNN). Example reference and query datasets and instructions are provided with the AltAnalyze software (DemoData) and online (https://github.com/AltAnalyze/cellHarmony-Align/tree/master/sample_data).

## 1.4 Combining ICGS Datasets

As an optional reference, cellHarmony can merge multiple ICGS outputs for diverse datasets (cellHarmonyMerge function). This method is recommended for cases in which users want to combine and compare multiple scRNA-Seq datasets simultaneously. The combined analysis uses the union of all supplied marker genes, averaged similar-cell profiles, and a separate file with all combined and ordered cells. To produce these results, the cellHarmonyMerge function: 1) selects all unique marker genes from the collectively provided set of inputs, 2) imports the expression data for these genes from the original complete gene matrix (e.g., AltAnalyze ExpressionInput "exp." file) and converts these to log2 values, 3) computes centroid expression for cells within the same identified cluster for all marker genes, 4) averages similar centroids "clusters" based on all pairwise centroid comparisons (Pearson correlation>0.9, default) to produce merged centroids, 5) filters the combined dataset to only include genes with non-zero values for all columns, 6) re-clusters the new reference centroids (HOPACH clustering) to produce a re-ordered matrix of reference cell-population centroids, 7) identifies and ranks marker genes for each of the combined clusters (MarkerFinder function, AltAnalyze) and 8) outputs a cell-level output for cellular barcodes corresponding to the cluster centroids for the final marker genes. The resultant all-cells reference text file can be used for downstream cellHarmony analyses. As an example, we have applied this workflow to 35 distinct cell populations we identified from bone marrow RNA-Seq generated by the Human Cell Atlas project, following independent ICGS analyses of each independent donor (1). This function can be run as:

```
$ python AltAnalyze.py --species Mm --platform RNASeq --cellHarmonyMerge yes --
centerMethod mean --correlationCutoff 0.9 --output $outpath --input $input1 --input
$input2 --input $input3 --input $input4
```

## 1.5 Programmatic Options and Parameter Tuning

When cellHarmony is run from the command-line, it requires specifying the following the user defined options: 1) the cell-alignment correlation cutoff (rho>0.4, default), 2) variable marker genes, 3)

differential expression p-value threshold (pval<0.05, default), 4) differential expression fold-change threshold (fold>1.5, default) and 5) FDR adjustment (adjp=True, default).

The code can be run as an independent python script in an alignment-only "rapid-mode" (https://github.com/AltAnalyze/cellHarmony-Align) or through the full cellHarmony programmatic or graphical user interface. When run in "rapid-mode", the user can modify multiple parameters for community clustering including the number of nearest neighbors (default =10) and number of trees for Annoy (default = 100), at which level to cut what level to cut the Louvain clustering dendrogram (default = 0), the minimum correlation for reporting a matching cell (default = -1), and the genes to consider for analysis. To optimize speed and memory utilization, h5 files are recommended. On a Mac OSX laptop with 16GB of RAM (2.9 GHz Intel Core i7), alignment of ~2,100 cell query cells to a ~3,800 cell reference completes in under a minute (https://github.com/AltAnalyze/cellHarmony-Align/tree/master/sample_data). For samples with weak heterogeneity (few community clusters) but many cells, the analysis time can be slower, (~10,000 cell query to a ~13,000 cell reference completing in 29 minutes with ~325MB maximum RAM used, with only 31 and 43 communities detected). Other alignment options present in the software include all cell-by-cell or cell-by-centroid alignments using k-nearest neighbor classification (KNN)(K=1), although community clustering is recommended (default). When applying these alternative comparisons, the nearest neighbor of a query cell profile is assigned to its best match in the reference set based on all possible cell-cell Pearson correlations.

The major determinants of which options to use are the experimental platforms compared (e.g., Fluidigm versus sci-CAR), increased or decreased data sparsity, and poorly detected heterogeneity within clusters. We recommend these parameters be tuned based on the following criteria: A) decreased or no fold-change threshold for highly sparse transcriptional data with excessive dropouts, B) use of non-adjusted moderated t-test for highly sparse data, C) use of an alternative ICGS/Seurat reference for highly sparse dataset with weakly detected heterogeneity, D) decreased correlation threshold when comparing highly dissimilar scRNA-Seq protocols/platforms, and E) increased correlation threshold in datasets where contaminating cells/doublets are present in the query or reference. No clear difference has been observed in our benchmarking of different ICGS clustering outputs (Euclidean versus Cosine) or use of marker genes in *a priori* defined clusters (see Mouse Cell Atlas example). Furthermore, if questionable alignments occur, the user is recommended to reverse the query and reference and re-run cellHarmony to determine whether cell or population predictions significantly differ (requires ICGS analysis of the new reference).

## 1.6 Output Files

As output, cellHarmony produces multiple tabular and visualization results, depending on the type of reference dataset supplied (full expression matrix versus pre-filtered). The initial outputs of cellHarmony are: 1) final association z-score matrix derived from the Pearson correlation coefficients for all cells to the *CellClassification/CellClassification.txt* file (labels optional), 2) expression matrix in which each cell is placed adjacent to its best match (*cellHarmony/heatmap* folder), 3) query-only cell matrix with cells ordered and annotated according to the classification (*cellHarmony/OtherFiles* folder), 4) gene expression heatmaps of the expression matrices (*cellHarmony/heatmap* folder), 5) cell frequency and gene expression difference bar charts (*cellHarmony/cell-frequency-stats.pdf*), 6) statistical differences in the frequency of aligned cell populations between reference and query samples (*cellHarmony/cell-frequency-stats.txt*), 7) statistically significant genes for all comparisons and associated values (*cellHarmony/DifferentialExpression_Fold_1.5_adjp_0.05*), 8) UMAP projection of the query and reference cells combined (*cellHarmony/DifferentialExpression_Fold_1.5_adjp_0.05*), 9) Pattern ordered heatmap with of fold differences in all compared cell populations (global, regional, local) with enriched Pathway Commons or transcription factor binding site gene-sets (*cellHarmony/OrganizedDifferentials.txt*), and 10) network plots and gene interaction lists (*cellHarmony/networks*). The log2-normalized expression profiles for both the reference and query are displayed as a combined heatmap, in the reference gene and cell order, with the query inserted alongside each cellHarmony match to assess their relative similarity. The frequency of cells present or absent from the query in each cell population is further reported and statistically quantified using a Fisher exact test to allow for the assessment of the lineage impact with cellular, molecular, or genetic perturbation in the query.

## 1.7 Evaluation Datasets

The following datasets were selected for evaluation within cellHarmony as they contained defined populations or have well-described cellular biology. Associated analysis scripts, input data files, and results can be obtained at https://www.synapse.org/#!Synapse:syn18500191.

Tabula Muris Mouse Cell Atlas (MCA): Gene by cell matrices for all SMART-Seq2 and 10x Genomics datasets were downloaded from GEO (GSE109774) and processed in ICGS version 2 using the software default options. Tissues samples with replicates were combined following counts normalization and log2 adjustment in AltAnalyze (SMART-Seq2 = import_scripts/combineCSV.py and import_scripts/CountsNormalize.py, 10x Genomics = import_scripts/ChromiumProcessing.py script) and jointly analyzed (import_scripts/MergeFiles.py script) (https://github.com/nsalomonis/altanalyze). Annotations for each MCA cell were obtained from the study authors

(https://github.com/czbiohub/tabula-muris/tree/master/00_data_ingest/03_tissue_annotation_csv). For comparative algorithm evaluation, no gene filtering was applied.

Transitional cell-states in bone marrow progenitor singlets: A dataset comprised of 383 hematopoietic bone marrow progenitor cells with high-confidence assigned cell-types and singlet-restricted profiles, validated via microfluidics cell capture imaging, was obtained from the GEO database along with the published ICGS unsupervised clustering results (GSE70245). Associated AML scRNA-Seq and bulk RNA-Seq were obtained from (GSE77849) and processed using the software RSEM to match the scRNA-Seq.

Murine Myocardial Infarction (MI): A previously unpublished scRNA-Seq dataset from a mouse model of myocardial infarction and sham surgery was produced for evaluation of cell-state specific transcriptomic differences (see Single-Cell RNA-Sequencing). This sequencing data, expression files, and metadata have been deposited in the open-access Synapse database (https://www.synapse.org/#!Synapse:syn18516494/files/). Count matrices were filtered to cells having a minimum of 200 genes expressed, proportion of mitochondrial genes < 0.25 and >= 400 UMIs (Sham n=13,858; MI n=11,240). Standard Seurat processing was conducted, including log-normalization, regressing out nUMI, mitochondrial proportion and cell cycle indicators (proportion of histone and Seurat G2/M transcripts), and scaling. Predicted cell doublet profiles were excluded from each dataset after selecting the union of doublet predictions from the softwares DoubletDecon and Scrublet (2,3). The application of the Seurat 3 integration procedure began with these objects. For the evaluation of cellHarmony, the Seurat cellHarmony processing script (http://altanalyze.org/cellHarmony/) was used to convert the Sham sample expression matrix, cell-state marker genes and cellular barcode clusters to an AltAnalyze compatible heatmap.

Human Acute Myeloid Leukemia Datasets. Data from two separate patient-matched AML studies were analyzed. The first study was a diagnostic and relapse sample from a patient with erythro-leukemia (p27) and post-transplantation biopsy profiled using the 10x Genomics Chromium platform (version 1 chemistry), obtained from the 10x Genomics website (https://support.10xgenomics.com/single-cell-gene-expression/datasets/1.1.0/aml027_pre_transplant, https://support.10xgenomics.com/single-cell-gene-expression/datasets/1.1.0/aml027_post_transplant). The data were processed from the supplied sparse-matrix input files in AltAnalyze using ICGS version 2 to obtain the post-transplant ICGS-NMF heatmap text file as the reference for cellHarmony. A second AML scRNA-Seq time-course was obtained from GEO (GSE116481) as a combined gene-counts file and pre-processed using the AltAnalyze

import_scripts/CountsNormalize.py python script. The day 0 diagnostic blood sample was used as the reference for cellHarmony following ICGS version 2 analysis. To identify less restricted gene expression differences at Day 2 and Day 4 compared to Day 0, cellHarmony was run without performing a p-value FDR adjustment and fold > 1.5. As a bulk RNA-Seq comparator, raw sequencing data from the Leucegene AML consortium (GSE49642, GSE52656, GSE62190, GSE67040) were downloaded and pseudo-aligned to the Ensembl 72 transcriptome using Kallisto. These samples were combined with healthy CD34+CD45RA- cord-blood samples from the same laboratory (GSE48846).

Human simulated RNA-Seq. Human HEK293T cells processed by 10X Genomics (http://support.10xgenomics.com/single-cell-gene-expression/datasets/2.1.0/hgmm_12k) were separated from mouse NIH3T3 cells and known doublets (mixed-species data). The dataset consists of genes expression (UMIs) profiles for cell captures categorized as human (n=6,164), murine (n=5,915) or mouse-human multiplet (n=741). These data were used for the evaluation of differential expression algorithms specifically in the HEK293T cells since they represent a presumably homogenous cell population.

## 1.8 Single-Cell RNA-Sequencing

Acute myocardial infarction (MI) was modeled in C57BL6/J wild type (WT) 8-10-week-old male mice (The Jackson Laboratories and confirmed via echocardiographic analysis, similar to previously described (4). A left thoracotomy was performed via the fourth intercostal space and the lungs retracted to expose the heart. After opening the pericardium, the left anterior descending coronary artery was ligated with 7-0 silk suture approximately 2 mm below the edge of the left atrial appendage. Ligation was considered successful when the anterior wall of the left ventricle turned pale. The lungs were inflated by increasing positive end-expiratory pressure and the thoracotomy site closed in layers with 6-0 suture. Animals were maintained on a 37 °C heating pad until recovery and for 2 h after surgery. Another group of mice underwent sham ligation, with a similar surgical procedure without tightening the suture around the coronary artery. Mice with an estimated pressure gradient across the aortic constriction below 40 mmHg were not included in the experiments. Hearts were collected at 14 days post-Sham surgery (n=4, pooled) or MI (n=1), perfused with ice-cold PBS to remove red blood cells followed by perfusion with 50 mM KCl to arrest the heart in diastole and then fixed for 4 hours in freshly prepared 4% PFA at 4 °C, rinsed with PBS and cryoprotected in 30% sucrose/PBS overnight before embedding in OCT (Tissue-Tek). DropSeq was performed as previously described (5). The quantity and quality of cDNA was measured using an Agilent Bioanalyzer hsDNA chip. To generate a library cDNA was fragmented and amplified (12 cycles) using the Nextera XT DNA Sample prep kit with three separate reactions of 600, 1,200 and 1,800 pg input cDNA. The libraries were pooled and purified twice using 0.7X volume of SPRIselect

beads. The purified libraries were quantified using an hsDNA chip and were sequenced on an Illumina HiSeq 2500 using the sequencing parameters described in the DropSeq protocol. Reads were aligned to the mm10 mouse genome using Bowtie2 (6) and tagged with the gene name of the overlapped exon. Gene reads were counted by unique UMIs per cell and an expression matrix was created. All animal procedures were performed and approved according to the Department of Laboratory Animal Medicine and the University Committee on Animal Resources at Cincinnati Children's Hospital Medical Center. scRNA-Seq data was deposited in Synapse (https://www.synapse.org/#!Synapse:syn18516494/files/).

## 1.9 Evaluation Details for cellHarmony

cellHarmony Evaluation Parameters: ICGS was run using AltAnalyze version 2.1.3 from input read counts or normalized count matrices using the software default options (7). The selected references for cellHarmony were produced and tested: R1) ICGS version 2.0 primary output file (ICGS-NMF/FinalMarkerHeatmap_all.txt), R2) intermediate ICGS output (ICGS/Guide-3 result), R3) marker profiles from previously derived cell clusters (supervised MarkerFinder analysis), or R4) the cellHarmonyMerge MarkerFinder output. ICGS outputs were produced using the default software options. Seurat-CCA was run as described above for the heart failure model analysis. For the Tabula Muris evaluation, gene-sets were tested for R1 and R3 (10x Genomics vs. SMART-Seq2) and R4 (10x Genomics test vs. training). For all Tabula Muris evaluations, no similarity cutoff was supplied, since cells were restricted to those with common labels in both the SMART-Seq2 and 10x Genomics datasets (46 Cell Ontology labels). No Pearson correlation cutoff was used for the mouse AML bone marrow analyses. For all other analyses, the default Pearson correlation threshold was applied. For differential expression analyses, the default options were applied with the exception of the mouse AML and human AML sample p27 comparisons, in which a more stringent fold change was applied (2-fold change), as these datasets had few cells overall and few cells aligned to most cell populations. A non-adjusted moderated t-test was applied for the Venetoclax treatment patient data to increase the detection of common gene expression differences during treatment.

Benchmarking Differential Expression Statistical Methods:

*Simulation*: Simulated single-cell sequencing data based on the human HEK293T cells from a publicly available 10X Genomics Mouse-Human mixed dataset were generated using the Bioconductor package splatter (8). Parameters for the simulated data were estimated using the splatEstimate function and 5 replicate count matrices with each of 50 and 100 cells per group were generated using the splatSimulate function (de.prob=0.05, de.facLoc=1, de.downProb=0.25). Rows (features) with all 0 entries were removed from count matrices. Expression matrices were computed by converting the count matrices to

counts-per-ten-thousand and applying the log2( +1) transformation. In addition to the empirical Bayes method implemented in cellHarmony, three high-performing methods identified in a review of single-cell differential expression analysis methods (9) were evaluated using these datasets: the Wilcoxon rank sum test, MAST (10) and SCDE (11). The eBayes, Wilcoxon and MAST analyses were supplied with expression matrices (MAST including the cellular detection rate covariate); SCDE with count matrices. The Type I error rate was calculated as the proportion of features for which the raw p-value was <0.05 among features with true log2-fold-change equal to 0. The false discovery rate (FDR) was calculated as the proportion of features with true log2-fold-change equal to 0 among features with FDR-adjusted-p-value <0.05. Statistical power curves were constructed to show the proportion of raw and FDR-adjusted p-values <0.05 by splatter differential expression log2 fold change parameter. Error rates and power curves were calculated for each replicate/group size/tool combination and for group size/tool combinations aggregated across replicates. Differential gene expression estimates from the software cellHarmony (empirical Bayes), SCDE and MAST were compared using bulk RNA-Seq as a control. Bulk RNA-Seq T-cells and B-cells (GSE51984) were aligned to the human genome (hg19) with the program STAR and analyzed using AltAnalyze to identify differentially expressed genes (DEGs) with an FDR corrected p<0.05. Single-cell RNA-Seq from human peripheral blood mononuclear cells (PBMCs) was downloaded from the 10x Genomics website (https://support.10xgenomics.com/single-cell-vdj/datasets) and processed in AltAnalyze with the ICGS algorithm to identify a CD8+ T-cell population and B-cell population. These data were compared in SCDE, MAST and AltAnalyze. For genes identified in the bulk and single-cell comparisons with a fold change in the same direction, DEGs were compared to calculate sensitivity and specificity.

**Details for Evaluated External Algorithms:**

scmap: scmap applies label projection from a reference dataset to a test dataset, by considering the agreement between multiple cell-similarity metrics. scmap was applied to two separate evaluation datasets: 1) 10x Genomics (47k) query cells compared to SMART-Seq2 (6k) reference and 2) 10x Genomics (24k test) query and 10x Genomics (24k training) reference. Both the query and reference were loaded as a "SingleCellExperiment" object which the scmap library uses as a scaffold. The "selected features" option was used to restrict the analysis to 500 variable genes for each dataset, based on expression and dropout-based rate. Clustering was performed using the "indexCluster" method, and projection was carried out using the standard scmap mapping algorithm, as outlined in the vignettes.

conos: conos (version 1.0.0) and pagoda2 (version 0.1.0) were obtained from https://github.com/hms-dbmi/conos and https://github.com/hms-dbmi/pagoda2 on April 2, 2019, and installed and run using R

version 3.5.0 on a linux computing cluster. We followed the online vignette to pre-process the two MCA sample count matrices with the basicP2proc (pagoda2) function (min.cells.per.gene=0, n.odgenes=2e3) and create a conos object. Pan-sample communities were found using the function findCommunities with default settings (method=leiden.community, resolution=1). The function propagateLabels was then applied, again with default settings.

CHETAH: CHETAH version 1.0.3 was installed from https://bioconductor.org/packages/release/bioc/html/CHETAH.html on July 10, 2019 and was run in the RStudio (version 1.1.447) environment for R (version 3.6.0) on a MacBook (High Sierra OS). We followed the online vignette to prepare SingleCellExperiment (SCE) objects for the two MCA samples. Following author recommendations, fitting a classifier for the (full) SMART-seq dataset and evaluating it with SMART-seq dataset was performed with a range of combinations of the parameters n_genes (500, recommended for sparse data; and 1000), subsample (T & F-default) and threshold (0, forcing a training dataset label onto every query cell; 0.10, the default value; 0.05, an intermediate value). The best training dataset performance (SMART-Seq2 only, not compared to 10x Genomics) was obtained with n_genes=1000 and threshold=0; changing the subsample option had negligible effect. The test of the 10x Genomics query onto the SMART-seq reference was run with n_genes=1000, subsample=F and threshold=0.

singleCellNet: singleCellNet version 0.3.1 was obtained from https://github.com/pcahan1/singleCellNet on July 10, 2019 and installed and run using R version 3.5.0 on a linux computing cluster. We followed the online vignette to load the count matrices for the two MCA samples. The subsequent steps of splitting the training data through assessment of the classifier were conducted as provided in the vignette, with the same parameter values except for setting dThresh=0 (default value) when transforming the training data and setting nRand=2 (in effect preventing the creation of an unknown/unclassified group as part of the classifier). The subsequent propagation of reference cell labels to the query dataset used default parameter settings, again with the exception of setting nRand=2.

Seurat: Seurat (development version 3.0.3.9015) was obtained from https://github.com/satijalab/seurat on July 22, 2019 and was run in the RStudio (version 1.1.447) environment for R (version 3.6.0) on a MacBook (High Sierra OS).

*Label projection with the Mouse Cell Atlas datasets* - Seurat objects were prepared for each the SMART-seq and 10X datasets, including the SMART-seq cell-type annotations provided by the study authors. We

followed the Integration and Label Transfer (Standard Workflow) vignette available at https://satijalab.org/seurat/v3.0/integration.html, applying the functions NormalizeData, FindVariableFeatures, FindTransferAnchors and TransferData with parameter values as per the vignette.

*Dataset Integration with Murine Myocardial Infarction datasets* - Starting Seurat objects for the Sham and MI samples were prepared as described in **Evaluation Datasets**. The integrated analysis followed the "Stimulated vs. Control PBMCs" vignette at https://satijalab.org/seurat/ applying the functions NormalizeData, FindVariableFeatures, FindIntegrationAnchors and IntegrateData with number of PCs increased from 20 to 30. Post-integration clustering was performed as outlined in the vignette and genes identified as differentially expressed using the FindConservedMarkers function to identify cell types.

Garnett: Garnett version 0.1.4 was installed from https://github.com/cole-trapnell-lab/garnett on July , 2019 and was run in the RStudio (version 1.1.447) environment for R (version 3.6.0) on a MacBook (High Sierra OS). We followed the online vignette to prepare CellDataSet (CDS) objects from count matrices and a formatted marker file (from ICGS output). The check_markers function (db="none", all other parameters with default values) was used to identify and remove markers for which the marker score could not be computed or that were classified as ambiguous (program determined score cutoff = 0.25). This filtering resulted in four cell-types without a marker. Alternative workflows that preserved markers for all cell-types were tried, but additional warnings appeared indicating that insufficient numbers of cell-type representative cells for some cell types were identified, leading to inability to build/train the classifier.

Reference Component Analysis (RCA): RCA associates individual cells with curated reference gene expression profiles through a correlation analysis., followed by a built-in method to define cell clusters (12). As RCA does not come with the inherent capability to include external references, only the three references utilized in their manuscript, modifications to the code are required to compare the algorithm performance to that of other alignment methods. Hence, users must make modifications to the RCA source code to allow for the addition of external references and the export of values required for assessment of accuracy, specifically: 1) The "sysdata.rda" file must be and loaded into R Studio, and 2) A new external reference, consisting of the described centroids and marker genes, must be inserted as the third reference dataset in the GlobalPanel list. ARI can be calculated for the results of RCA, but not accuracy, as the method does not allow for direct label projection. See the synapse data repository for an example.

# 2 Supplementary Figures

## 2.1 Supplementary Figure S1

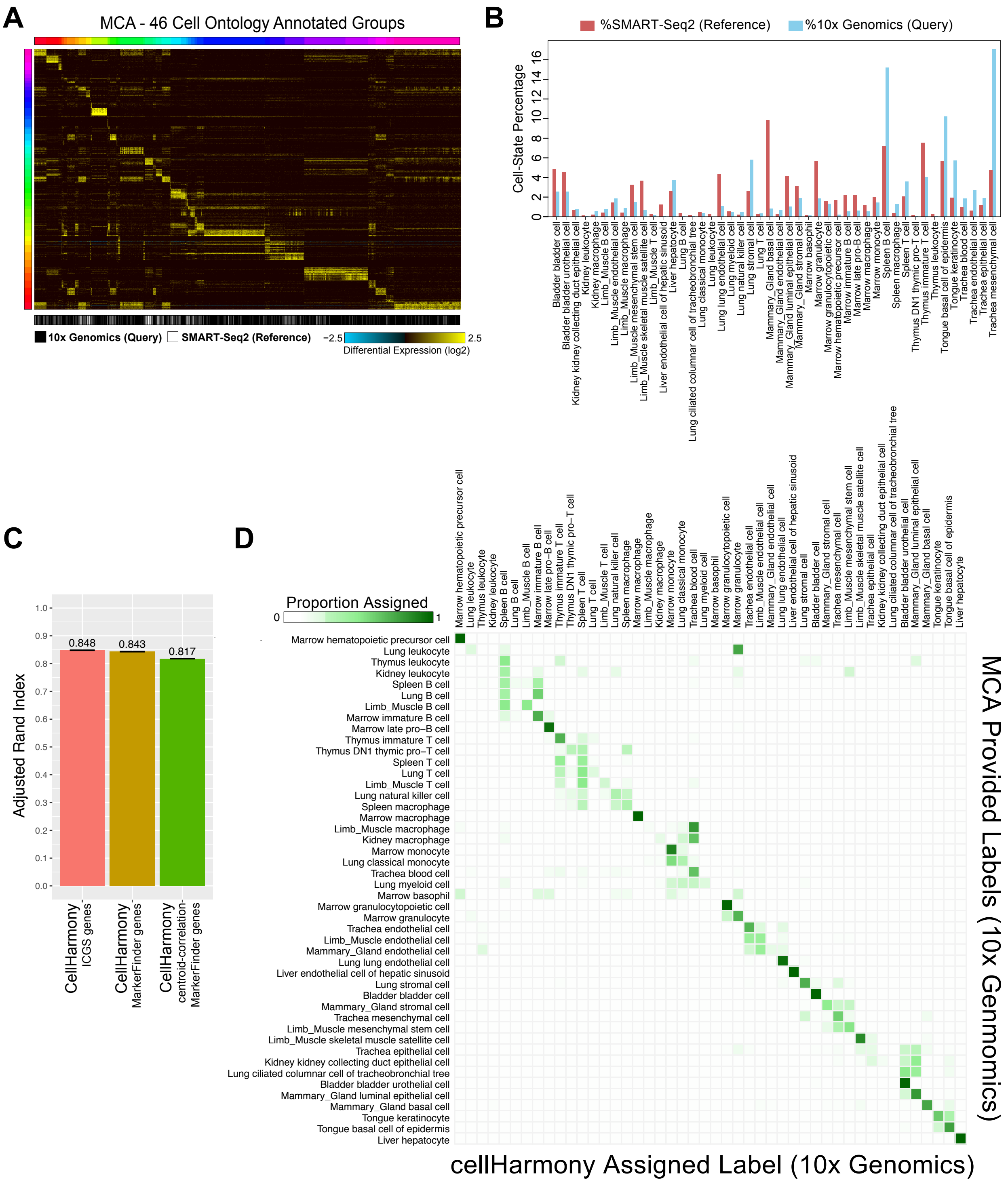

**Figure S1. Alignment of previously defined multi-tissue mouse cell atlas clusters with cellHarmony**. A) cellHarmony alignment results as a combined heatmap for a large mouse cell atlas (Tabula Muris), corresponding to >47,000 10x Genomics scRNA-Seq profiled cells from 12 tissues (query) against >6,000 annotated SMART-Seq2 scRNA-Seq profiles from the same tissues. Cells in both datasets were restricted to those which had common Cell Ontology labels across technologies, within each cell-type (e.g., Lung Macrophage labeled from SMART-Seq2 and Lung Macrophage labeled from 10x Genomics). Gene clusters were defined based on the Cell Ontology labels from the SMART-Seq2 dataset, with markers derived using the MarkerFinder function within AltAnalyze (top 60 reported markers for each annotated tissue by cell-type). At the top of the heatmap are clusters denoted by different colors and on left are genes associated with those clusters. Although some cell-types (e.g., B-cells) will have near identical transcriptomes in different cell-types, these were still kept as independent clusters for alignment, but will not always have unique marker gene clusters. Cells corresponding to the two different technologies are displayed below the heatmap (not all white and black lines will be visible). B) Percentage of cells for each cell-population that were defined by the original study authors (SMART-Seq2) or by cellHarmony alignment (10x Genomics). Note, for each technology, different isolation methods were employed (FACs in SMART-Seq2 and unbiased in 10x Genomics). C) Comparison of Adjusted Rand Index agreement values cellHarmony runs using either variable genes from an unsupervised analysis of the SMART-Seq2 dataset (ICGS version 2) or marker genes identified from the pre-defined 46 Cell Ontology defined populations in the SMART-Seq2 dataset (MarkerFinder algorithm). Comparable results are shown using the average expression profiles for the 46 cell types (centroids) using the centroid correlation option in cellHarmony. D) Specificity of alignments from 10x Genomics cells (50% test set) against 10x Genomics cells (50% training set) for the original author denoted Cell Ontology terms. Increased saturation of the squares indicates a higher proportion of assignments, with the darkest green representing 100% assignment in that category. Note, B-cell, T-cell and leukocyte ambiguity across tissues, as expected.

## 2.2 Supplementary Figure S2

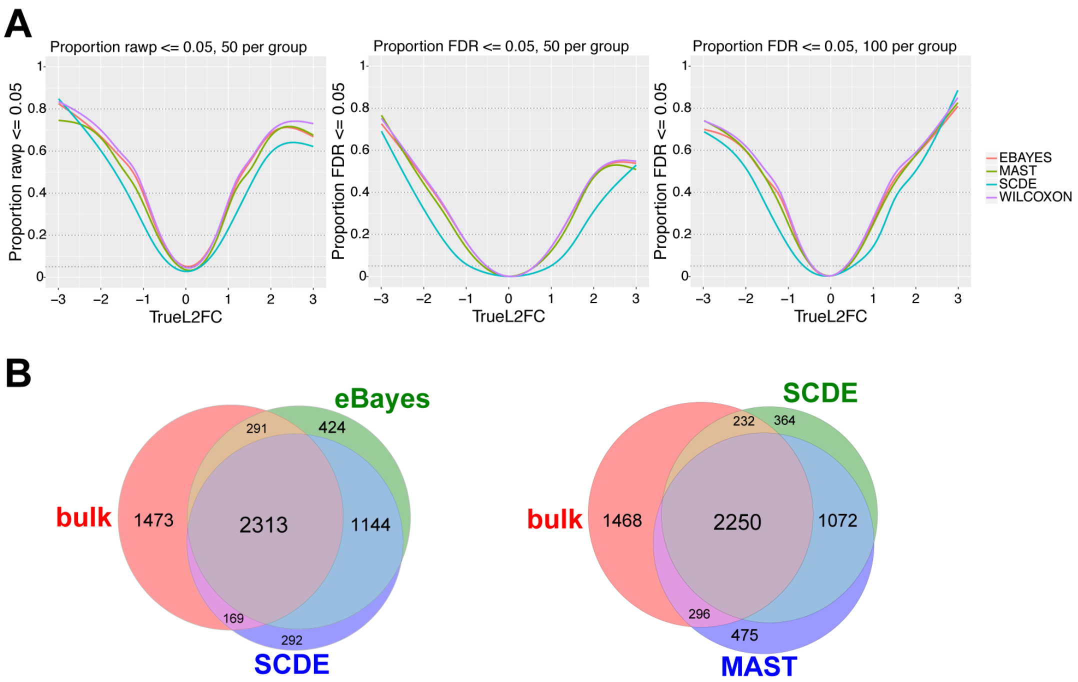


**Figure S2. Evaluation of different algorithms for single-cell differential gene expression analysis.** A) Statistical power curves for simulated RNA-Seq data indicate the proportion of tests (features) with unadjusted or FDR-adjusted p-values ≤ 0.05 over a range of log2 fold-change values. Simulated data was generated using the R package "splatter" with 50 or 100 cells for each of the two groups (composite of 5 simulated datasets). B) Overlap of differentially expressed genes (DEGs) from T-cells versus B-cells profiled either by bulk RNA-Seq (benchmark data) or scRNA-Seq predicted using the MAST or SCDE testing procedure or empirical Bayes t-test (eBayes) (see Supplemental Information).

## 2.3 Supplementary Figure S3

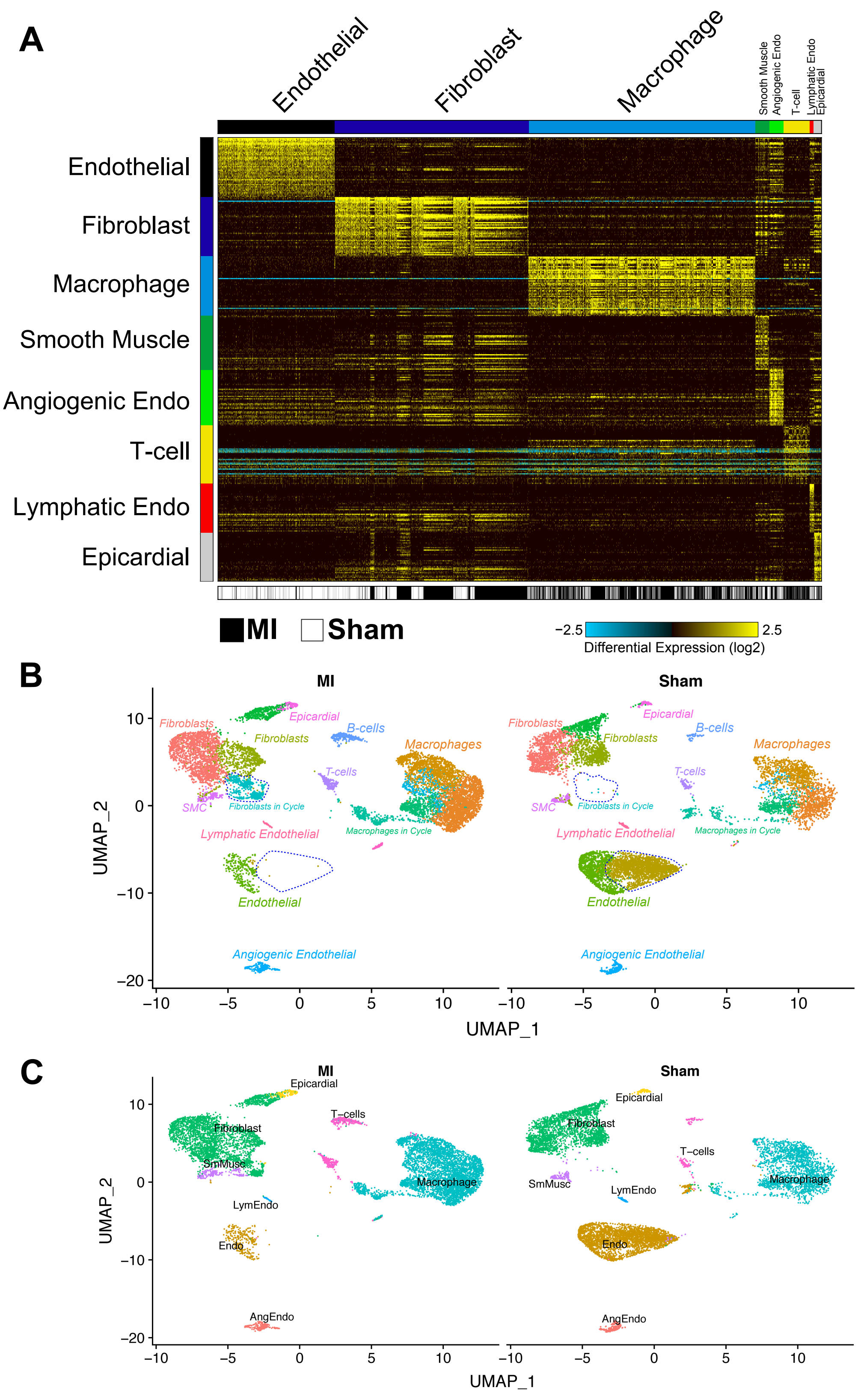

**Figure S3. Analysis of lineage priming in heart ischemia by cellHarmony and Seurat3.**

A) Default output cellHarmony combined heatmap of the MI (query) and Sham (reference) alignments, with cell-type labels derived from AltAnalyze gene-set enrichment analysis for the obtained Sham Seurat clusters. B) Joint-alignment analysis results (Seurat3 multi-dataset integration) for healthy (Sham) and diseased (myocardial infarction: MI) viewed as a UMAP plot for the two sample groups. Clusters are annotated based on cellHarmony assigned labels in C and gene-set enrichment analysis (ToppFun). C) Indication of cellHarmony defined cell-type MI annotations (and the original Sham only Seurat clustering annotations) from **Fig. 3A** displayed in the Seurat UMAP graphs.

## 2.4 Supplementary Figure S4

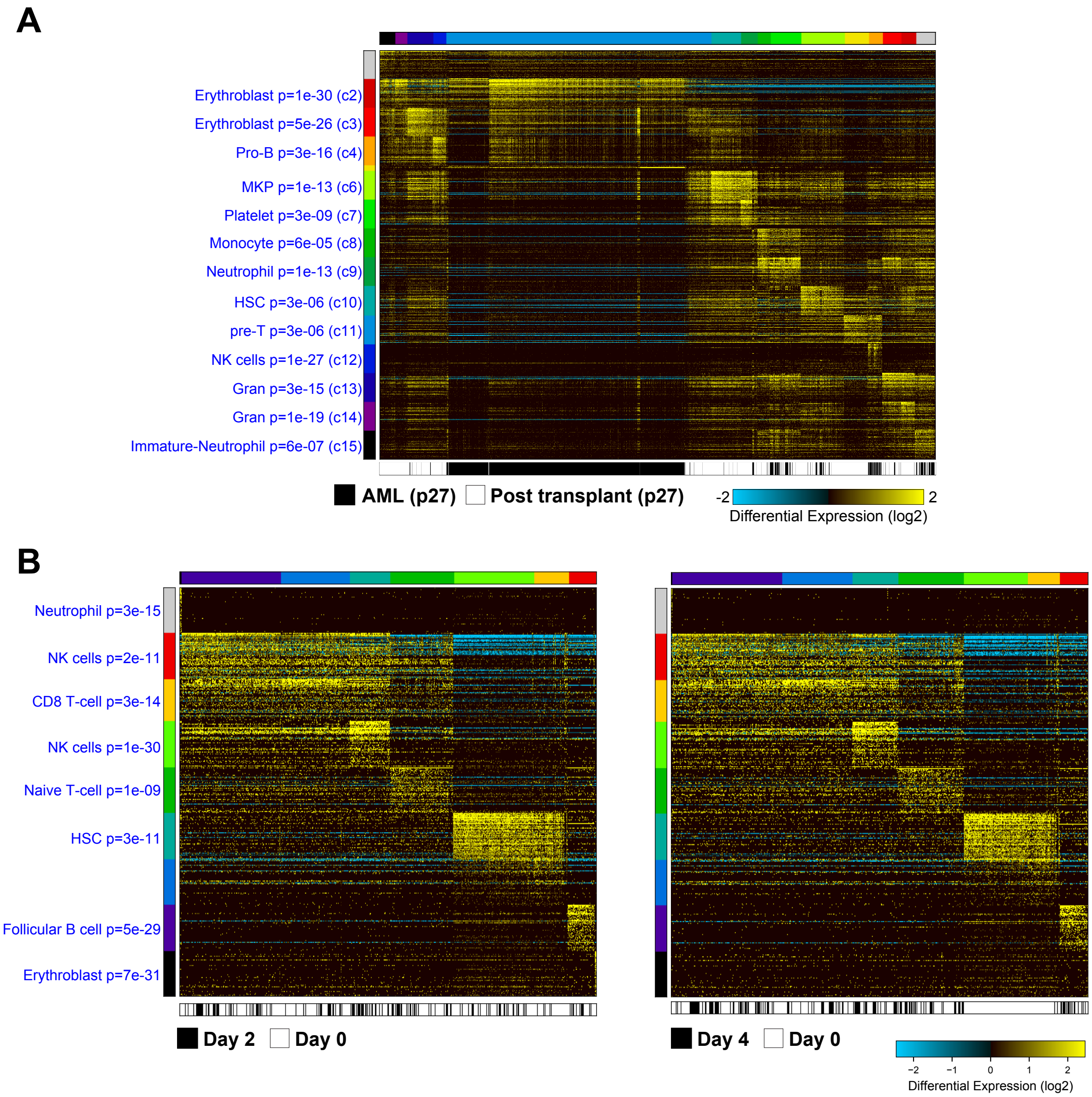


**Figure S4. cellHarmony comparison of acute myeloid leukemia patient samples overtime time.** A) cellHarmony heatmap human bone marrow mononuclear cells (BMMCs) from an AML patient at diagnosis (query) aligned to a post-transplantation biopsy (reference) from the same individual. Cell-type predictions are using input gene-sets from Hay et al. with AltAnalyze (derived human bone marrow cell population markers). Reference AML diagnosis cell populations were determined using ICGS-NMF. B) Similar heatmaps as in A, with peripheral blood cells from AML, 2 days (left) or 4 days (right) after the induction of chemotherapy (query) relative to Day 0 cells (reference). Reference AML Day 0 cell populations were determined using ICGS-NMF.

# 3 References


1. Hay, S.B., Ferchen, K., Chetal, K., Grimes, H.L. and Salomonis, N. (2018) The Human Cell Atlas bone marrow single-cell interactive web portal. *Exp Hematol*.
2. DePasquale, E.A.K., Schnell, D.J., Valiente, I., Blaxall, B.C., Grimes, H.L., Singh, H. and Salomonis, N. (2018) DoubletDecon: Cell-State Aware Removal of Single-Cell RNA-Seq Doublets. *bioRxiv*.
3. Wolock, S.L., Lopez, R. and Klein, A.M. Scrublet: Computational Identification of Cell Doublets in Single-Cell Transcriptomic Data. *Cell Systems*.
4. Duan, Q., McMahon, S., Anand, P., Shah, H., Thomas, S., Salunga, H.T., Huang, Y., Zhang, R., Sahadevan, A., Lemieux, M.E. *et al.* (2017) BET bromodomain inhibition suppresses innate inflammatory and profibrotic transcriptional networks in heart failure. *Sci Transl Med*, **9**.
5. Macosko, E.Z., Basu, A., Satija, R., Nemesh, J., Shekhar, K., Goldman, M., Tirosh, I., Bialas, A.R., Kamitaki, N., Martersteck, E.M. *et al.* (2015) Highly Parallel Genome-wide Expression Profiling of Individual Cells Using Nanoliter Droplets. *Cell*, **161**, 1202-1214.
6. Langmead, B., Trapnell, C., Pop, M. and Salzberg, S.L. (2009) Ultrafast and memory-efficient alignment of short DNA sequences to the human genome. *Genome biology*, **10**, R25.
7. Olsson, A., Venkatasubramanian, M., Chaudhri, V.K., Aronow, B.J., Salomonis, N., Singh, H. and Grimes, H.L. (2016) Single-cell analysis of mixed-lineage states leading to a binary cell fate choice. *Nature*, **537**, 698-702.
8. Zappia, L., Phipson, B. and Oshlack, A. (2017) Splatter: simulation of single-cell RNA sequencing data. *Genome biology*, **18**, 174.
9. Soneson, C. and Robinson, M.D. (2018) Bias, robustness and scalability in single-cell differential expression analysis. *Nature methods*, **15**, 255-261.
10. Finak, G., McDavid, A., Yajima, M., Deng, J., Gersuk, V., Shalek, A.K., Slichter, C.K., Miller, H.W., McElrath, M.J., Prlic, M. *et al.* (2015) MAST: a flexible statistical framework for assessing transcriptional changes and characterizing heterogeneity in single-cell RNA sequencing data. *Genome biology*, **16**, 278.
11. Kharchenko, P.V., Silberstein, L. and Scadden, D.T. (2014) Bayesian approach to single-cell differential expression analysis. *Nature methods*, **11**, 740-742.
12. Li, H., Courtois, E.T., Sengupta, D., Tan, Y., Chen, K.H., Goh, J.J.L., Kong, S.L., Chua, C., Hon, L.K., Tan, W.S. *et al.* (2017) Reference component analysis of single-cell transcriptomes elucidates cellular heterogeneity in human colorectal tumors. *Nature genetics*, **49**, 708-718.
